# Chemical basis for the recognition of trimethyllysine by epigenetic reader proteins

**DOI:** 10.1038/ncomms9911

**Published:** 2015-11-18

**Authors:** Jos J.A.G. Kamps, Jiaxin Huang, Jordi Poater, Chao Xu, Bas J.G.E. Pieters, Aiping Dong, Jinrong Min, Woody Sherman, Thijs Beuming, F. Matthias Bickelhaupt, Haitao Li, Jasmin Mecinović

**Affiliations:** 1 Institute for Molecules and Materials, Radboud University, Heyendaalseweg 135, 6525 AJ Nijmegen, The Netherlands; 2 Department of Basic Medical Sciences, Center for Structural Biology, School of Medicine, Tsinghua University, Beijing 100084, China; 3 Department of Theoretical Chemistry and Amsterdam Center for Multiscale Modeling, VU University, De Boelelaan 1083, 1081 HV Amsterdam, The Netherlands; 4 Structural Genomics Consortium, University of Toronto, 101 College Street, Toronto, Ontario, Canada M5G 1L7; 5 Schrödinger, Inc., 120 West 45th Street, New York, New York 10036 USA

## Abstract

A large number of structurally diverse epigenetic reader proteins specifically recognize methylated lysine residues on histone proteins. Here we describe comparative thermodynamic, structural and computational studies on recognition of the positively charged natural trimethyllysine and its neutral analogues by reader proteins. This work provides experimental and theoretical evidence that reader proteins predominantly recognize trimethyllysine via a combination of favourable cation–*π* interactions and the release of the high-energy water molecules that occupy the aromatic cage of reader proteins on the association with the trimethyllysine side chain. These results have implications in rational drug design by specifically targeting the aromatic cage of readers of trimethyllysine.

## Abstract (web summary)

A structurally diverse set of epigenetic reader proteins can recognize methylated lysine residues on histones. Here the authors show that recognition of trimethyllysine occurs through a combination of favourable cation–*π* interactions and the release of water molecules occupying the aromatic cages of reader proteins.

## Graphical abstract

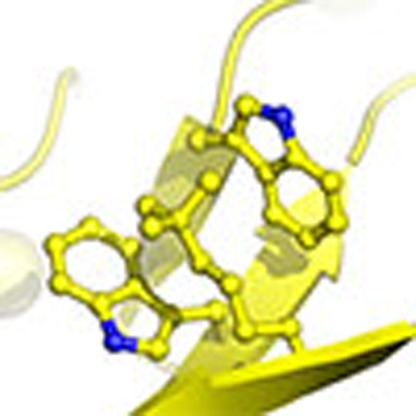

The positioning and chemical diversity of post-translational modifications on histone proteins orchestrate the structure and function of the eukaryotic chromatin^1^,^2^,^3^. One such modification is lysine methylation, which is associated both with gene activation and repression, depending on the type of histone and details of the sequence site^4^. The methylation of lysine residues of histone proteins is a dynamic process that is regulated by SAM-dependent histone lysine methyltransferases, FAD- or Fe(II)/2OG-dependent histone demethylases, and reader proteins (also known as effector proteins) that specifically recognize post-translationally modified lysines in histones and affect the downstream cellular processes^5^,^6^,^7^. Enzymatic lysine methylation can lead to the formation of monomethyllysine (Kme1), dimethyllysine (Kme2) and trimethyllysine (Kme3), with each methylation mark being specifically recognized by different classes of the interacting reader proteins^8^. Lower methylation states Kme1 and Kme2 are specifically read by 53BP1 tandem tudor domains, L3MBTL1 MBT repeats, G9a ankyrin repeats and ORC1 BAH domain, primarily via the cavity-insertion binding mode^9^,^10^,^11^,^12^,^13^. The constitution of the Kme1/Kme2 recognition site enables the specificity in two ways: the methylammonium group forms the energetically favourable hydrogen bonding and electrostatic interactions with the negatively charged aspartate or glutamate, allowing the methyl group to position towards the aromatic residues, and the narrow binding pocket sterically prevents the access of the bulkier Kme3. The highest methylation state Kme3 is specifically recognized by a structurally diverse class of reader proteins, including plant homeodomain (PHD) zinc fingers, WD40 repeats and members of the Royal superfamily (tudor domain, chromodomain and PWWP domain), in the surface-groove binding mode^8^,^10^. For the Kme3 reading modules, binding studies of histone peptides showed that binding affinities typically follow the trend Kme3>Kme2>Kme1>K (ref. 14). With the exception of ATRX ADD domain, most characterized reader proteins specifically recognize Kme3 through an aromatic cage that consists of 1–4 aromatic amino acids (Phe, Tyr and Trp) and/or one methionine^15^. Aromatic cages of several reader modules also contain negatively charged Asp or Glu residues. The positioning of the quaternary ammonium (Kme3) group inside the aromatic cage, as demonstrated by structural determination of several reader–Kme3 complexes, suggests that the specific readout process is primarily driven by cation–*π* interactions, although charge-independent interactions may also contribute to the overall binding^8^,^16^,^17^,^18^. Herein we report clear experimental and computational support for the chemical basis for the recognition of Kme3-containing histones by reader proteins. Our study reveals that the association between trimethyllysine and the aromatic cage of reader proteins is driven by energetically favourable cation–*π* interactions between the positively charged trimethyllysine and the electron-rich aromatic cage, and the trimethyllysine-mediated release of non-optimally structured water molecules that occupy the aromatic cages of reader proteins.

## Results

### Physical–organic chemistry approach

**Figure 1:**
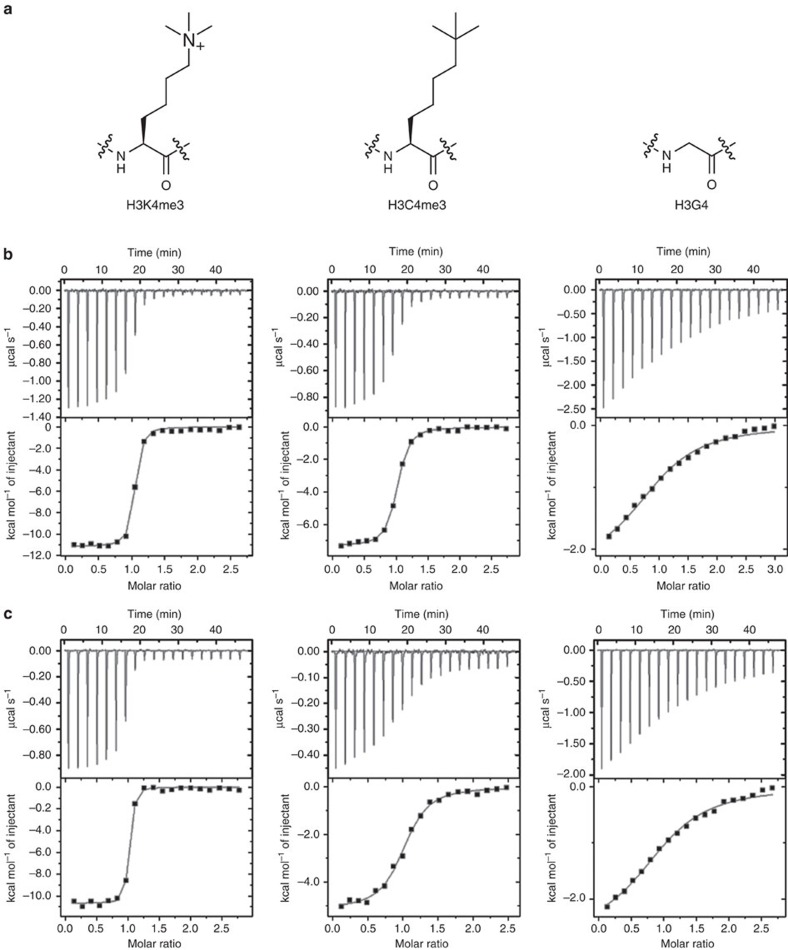
Thermodynamic analyses of binding. (**a**) Structures of the positively charged Kme3 and neutral Cme3 and G analogues; (**b**) ITC curves of 10-mer H3K4me3, H3C4me3 and H3G4 histone peptides binding to the JARID1A PHD3 domain; (**c**) ITC curves of 10-mer H3K4me3, H3C4me3 and H3G4 histone peptides binding to the TAF3 PHD domain.

Specific favourable binding of the positively charged side chain of Kme3 to the aromatic cage of reader proteins could, in principle, be a result of (i) favourable solute–solute interactions (cation–*π* and CH–*π* interactions), (ii) partial desolvation of the Kme3 side chain of histone tails (via the hydrophobic effect), and/or (iii) desolvation of the aromatic cage of reader proteins. To elucidate the underlying chemical basis for the recognition of natural Kme3 by reader proteins, we have carried out detailed comparative studies for binding of 10-mer histone peptides that contain the positively charged Kme3, its neutral carba analogue Cme3, and the glycine residue that lacks the entire side chain at the fourth position of histone 3 (that is, H3K4me3, H3C4me3 and H3G4; Fig. 1a). We have chosen the simplest uncharged Cme3 analogue to directly probe the involvement of the proposed cation–*π* interactions in reader–histone associations, because it has virtually the same size, shape and polarizability as the positively charged Kme3, but lacks the presence of the fixed positive charge^16^. Values for volumes of Kme3 (160.2 Å^3^) and Cme3 (158.2 Å^3^) indicate that, in the case that the binding mode is the same for both side chains, they should displace the same amount of water molecules from the protein site on binding. We have chosen the 10-mer H3G4 peptide to explore the importance of the entire side chain of Kme3 on association with reader proteins. The Kme3/Cme3→G substitution directly probes the significance of the potential displacement of water molecules that are localized inside the aromatic cage of reader proteins.

### Thermodynamic analyses of reader–histone association

We use isothermal titration calorimetry (ITC) to obtain full thermodynamic descriptions for binding of H3K4me3, H3C4me3 and H3G4 peptides to five representative reader proteins that specifically recognize H3K4me3 (the PHD zinc fingers of JARID1A, BPTF, TAF3 and the tudor domains of the Royal family of SGF29 and JMJD2A) (Table 1 and Fig. 1b,c)^19^,^20^,^21^,^22^,^23^. The five reader pockets are different in the aromatic cage composition and architecture, which allows us to examine the effect of individual constitution of the aromatic cage on binding differences. Comparative ITC experiments for the associations of H3K4me3 and H3C4me3 showed that: (i) the positively charged H3K4me3 binds 2–33-fold stronger than the neutral H3C4me3 to 4 out of 5 reader proteins that contain Trp as part of the aromatic cage (JARID1A, TAF3, BPTF and JMJD2A; Table 1); (ii) association of the Kme3 side chain with the aromatic cage is on average about 4.3 kcal mol^−1^ more favourable in enthalpy than the association of the neutral Cme3 group to the same cage; and (iii) association of the Kme3 side chain is about 3.1 kcal mol^−1^ less favourable in entropy than the association of the Cme3 group to the same aromatic pocket. Collectively, these data provide evidence for the presence of the favourable cation–*π* interactions in the natural readout process, as exemplified by the enthalpy-driven association of the naturally occurring Kme3 with the electron-rich aromatic cage of reader proteins. In contrast to other readers that contain at least one Trp residue, H3K4me3 and H3C4me3 bound to the tandem tudor domain of SGF29 with virtually indistinguishable thermodynamics of associations, indicating the lack (or at least a minor contribution) of cation–*π* interactions in the association of Kme3 by the Tyr/Phe-containing half aromatic cage of SGF29 (Table 1). This result is consistent with the well-established observation that the strength of cation–*π* interactions depends on the nature of the aromatic ring^24^,^25^,^26^,^27^,^28^,^29^,^30^,^31^,^32^. Studies on the related protein systems showed that Trp forms significantly stronger cation–*π* interactions with quaternary ammonium ions than do Phe or Tyr residues^24^,^25^. For SGF29, the electrostatic interactions between Kme3 and D266, and between the positively charged α-amino group of A1 and the H3A1 binding pocket importantly contribute to the overall binding affinity of H3K4me3 (refs 22, 33).

Negative values of the heat capacities (ΔC_p_) for binding of H3K4me3 and H3C4me3 to reader proteins were also determined by ITC. In all the cases examined, we observed more negative values for H3C4me3 than for H3K4me3: JARID1A–H3K4me3 −162±4 cal mol^−1^ K^−1^, JARID1A–H3C4me3 −182±3 cal mol^−1^ K^−1^; TAF3–H3K4me3 −142±7 cal mol^−1^ K^−1^, TAF3–H3C4me3 −171±8 cal mol^−1^ K^−1^; BPTF–H3K4me3 −103±6 cal mol^−1^ K^−1^, BPTF–H3C4me3 −145±7 cal mol^−1^ K^−1^ (Supplementary Fig. 1). These results are in agreement with the involvement of the classical hydrophobic interactions for binding of H3C4me3 to the aromatic cage of reader proteins; this suggests that entropy-driven (partial) desolvation of the Cme3 side chain contributes favourably to the binding affinity^34^,^35^,^36^,^37^. Binding of the uncharged Cme3 to the aromatic cage can additionally be attributed to the energetically favourable CH–*π* hydrogen bonding with a strong polarization component^38^,^39^.

We further examined the contribution of the entire Kme3 side chain to the overall binding associations with the aromatic cage of reader proteins. ITC data showed that binding of 10-mer H3G4 to all five reader proteins was dramatically reduced (>500-fold) when compared with binding of the H3K4me3 counterpart, highlighting the importance of the entire side chain in the complexation process. More detailed thermodynamic analyses were only possible with JARID1A and TAF3, because both proteins bind to the reference H3K4me3 peptide with *K*_d_ values in submicromolar range and the H3G4 peptide had sufficient residual affinity for ITC characterization (Fig. 1b,c): JARID1A–H3G4 (*K*_d_=88 μM, Δ*G*°=−5.5 kcal mol^−1^, Δ*H*°=−2.1 kcal mol^−1^, −TΔ*S*°=−3.4 kcal mol^−1^) and TAF3–H3G4 (*K*_d_=36 μM, Δ*G*°=−6.1 kcal mol^−1^, Δ*H*°=−2.5 kcal mol^−1^, −TΔ*S*°=−3.6 kcal mol^−1^). Overall, thermodynamic data revealed that (i) binding of the entire side chain of the Kme3 contributes about −4 kcal mol^−1^ (that is, about 40%) to the overall Gibbs binding free energy (Δ*G*°); (ii) favourable enthalpy provides a dominant contribution (∼−8.5 kcal mol^−1^) to the binding of the entire Kme3 side chain to the aromatic cage; and (iii) entropy of binding becomes more favourable (−TΔΔ*S*°=−4.5 kcal mol^−1^) for H3G4 relative to H3K4me3. In addition to thermodynamics results on H3C4me3, these results indicate that favourable cation–*π* interactions are not solely responsible for strong binding affinity of H3K4me3, but that other types of solute–solute interactions and reader/histone desolvation could also play an important role in the specific readout of Kme3.

### Structural determination of reader–H3C4me3 complexes

**Figure 2:**
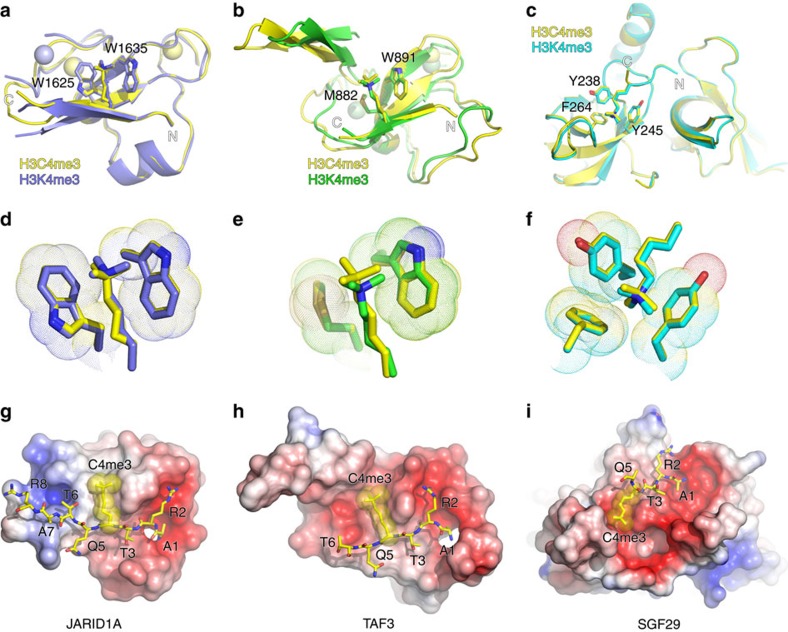
Structural analyses of reader–histone interactions. Structural superimposition of H3C4me3 and H3K4me3–bound complexes of (**a**) the JARID1A PHD finger, (**b**) the TAF3 PHD finger and (**c**) the SGF29 tandem tudor domains. Overall structures are represented in ribbon view with key residues highlighted in stick. In all panels, the H3C4me3 peptides and their complexes are coloured yellow, and the H3K4me3 counterparts are colour coded blue for JARID1A, green for TAF3, and cyan for SGF29. Small spheres, zinc ions. Close-up view of the reader pockets are shown in **d** for JARID1A, **e** for TAF3 and **f** for SGF29. Van der Waals surfaces of caging residues are depicted as dots. Electrostatic surface view of H3C4me3 complexes of (**g**) JARID1A, (**h**) TAF3 and (**i**) SGF29. Red and blue colours indicate negative and positive electrostatic potential, respectively. H3 peptides are shown in stick mode with C4me3 side chain overlaid with dotted van der Waals surfaces.

Having shown that the removal of the positive charge in Kme3 (as in the neutral H3C4me3) resulted in reduced binding affinity for most reader proteins due to less favourable enthalpy of binding, we aimed to rationalize these results in conjunction with structural analyses for reader–H3C4me3 complexes. We solved three X-ray crystal structures for complexes with JARID1A, TAF3 and SGF29 at 1.6–2.8 Å resolution (Fig. 2 and Table 2). All three reader–H3C4me3 structures clearly illustrated that the uncharged side chain of C4me3 is positioned well inside the aromatic cages of JARID1A, TAF3 and SGF29, virtually in the same binding mode as the positively charged Kme3 (Fig. 2a–c). The calculated average values of the root-mean-squared deviation for binding of ‘Cme3' and ‘Kme3'–aromatic cage pairs were: 0.124 Å for JARID1A, 0.261 Å for TAF3 and 0.108 Å for SGF29, respectively, suggesting essentially the same complexation mode engaging in aromatic pocket residues upon binding of neutral C4me3 (Fig. 2d–f). In all three complexes, the carba histone peptide binds to an electrostatically negative surface with the long C4me3 side chain positioned in a surface groove formed by the caging residues (Fig. 2g–i and Supplementary Fig. 2). On the formation of the JARID1A–H3C4me3 complex, the buried solvent accessible surface area (SASA) of C4me3 (hydrogen atoms added) is 160.6 Å^2^, which accounts for 38.8% of the total SASA of C4me3, as compared with Kme3 binding to JARID1A with a buried SASA of 163.8 Å^2^, which equals 39.5% of total SASA. Similar features have also been observed for binding of H3C4me3 and H3K4me3 to TAF3 with 48.3% buried SASA for H3C4me3 and 50.1% buried SASA for H3K4me3, and to SGF29 with 48.6 and 47.3% buried SASA for H3C4me3 and H3K4me3, respectively.

### Computational analyses in the gas and aqueous phase

Our aim is to elucidate the nature and selectivity of the non-covalent interactions between the aromatic cage that consists of two tryptophan residues of JARID1A (hereafter designated as TRP2 fragment) and the Kme3 versus Cme3 side chain of the histone peptide. To this end, we have quantum chemically characterized the energetics and bonding mechanism in two model complexes, using dispersion-corrected density functional theory at BLYP-D3BJ/TZ2P and COSMO for simulating aqueous solution, as implemented in the Amsterdam Density Functional (ADF) program^40^,^41^. The model complexes consist of those moieties of the JARID1A–H3K4me3 and JARID1A–H3C4me3 X-ray structures that give rise to the intermolecular interaction in the full reader–histone complexes (Supplementary Table 1). The chosen subsystems were terminated with one hydrogen at C_β_ of the Kme3 or Cme3 side chain and one hydrogen at each C_β_ of the TRP2 fragment. Thus, Kme3 and Cme3 fragments are fully optimized, both as isolated molecules and as molecular fragments in the complex with TRP2. To simulate the structural rigidity that is imposed by the protein backbone in the full protein system, the TRP2 fragment is kept frozen to the X-ray structure, both as a separate fragment and in the complexes. Geometries of the optimized model systems differ only very slightly from the X-ray structures.

Our computations show that, in line with experimental data, there is an energetic preference of ∼2 kcal mol^−1^ for the JARID1A–Kme3 over the JARID1A–Cme3 model complex with bond energies Δ*E*(aq) of −10.2 and −8.4 kcal mol^−1^, respectively (Table 3). The geometries of the two model complexes are similar, but NMe_3_^+^ in the JARID1A–Kme3 model is somewhat closer to the TRP2 tryptophan cage than CMe_3_ in the JARID1A–Cme3 model. The closest H–C distances between an NMe_3_^+^ H atom and a C atom of a tryptophan in the JARID1A–Kme3 model is 2.78 Å, while the same H atom is 3.38 Å away from the closest C atom of the other tryptophan. For comparison, the corresponding H–C distances in the JARID1A–Cme3 model are 3.16 and 3.15 Å (Table 3 and Supplementary Fig. 3). A characteristic difference in geometries comes from the conformation of Kme3 and Cme3. In the former, the chain of four carbon atoms has a zigzag conformation whereas, in the latter, this chain is U shaped.

Our bonding analyses reveal that the bond energies Δ*E*(aq) associated with the molecular recognition processes of Kme3 versus Cme3 in water are essentially identical with the corresponding instantaneous interaction energies Δ*E*_int_(aq) of −10.3 and −8.7 kcal mol^−1^, respectively. The reason is that complexation only very slightly changes the geometry of the Kme3 and Cme3 side chains as a result of which the associated deformation strain is negligible, that is, 0.1 and 0.3 kcal mol^−1^, respectively. The intrinsic preference for Kme3 over Cme3, that is, the interaction energy Δ*E*_int_ between the same structures but in the absence of the solvent, is even more in favour of the former with values of −27.6 and −10.9 kcal mol^−1^, respectively (Table 3). The significantly stronger interaction energy of Kme3 is, however, strongly attenuated by the desolvation incurred on binding, which is significantly more unfavourable for Kme3. Thus, solvent effects destabilize the JARID1A–Kme3 complex by +17.3, whereas the desolvation penalty in the JARID1A–Cme3 complex is only +2.2 kcal mol^−1^. The reason for this large difference can be attributed to the removal of solvent (desolvation) around the positive charge of the Kme3 side chain ammonium group. Note that the stronger binding in JARID1A–Kme3 causes a reduction in the bond distances (see above), resulting in a computed Pauli repulsion energy between closed shells that is +6.7 kcal mol^−1^ more repulsive for this more stable JARID1A–Kme3 complex.

The reason why the TRP2 unit interacts more favourably with Kme3 than with Cme3 becomes clear from our quantitative Kohn–Sham molecular orbital and energy decomposition analyses (EDA) of the interaction energy Δ*E*_int_ (Table 3)^42^. Interestingly, although dispersion Δ*E*_disp_ is the largest contributor to the reader–histone interaction, it contributes only 4.4 kcal mol^−1^ to the 16.7 kcal mol^−1^ difference in Δ*E*_int_ between JARID1A–Kme3 (−27.6 kcal mol^−1^) and JARID1A–Cme3 (−10.9 kcal mol^−1^; Table 3). Instead, the difference in stability between JARID1A–Kme3 and JARID1A–Cme3 mainly originates from the electrostatic (ΔV_elstat_) and orbital interaction (ΔE_oi_) terms that favour the complex with Kme3 by 9.6 and 9.4 kcal mol^−1^, respectively.

**Figure 3:**
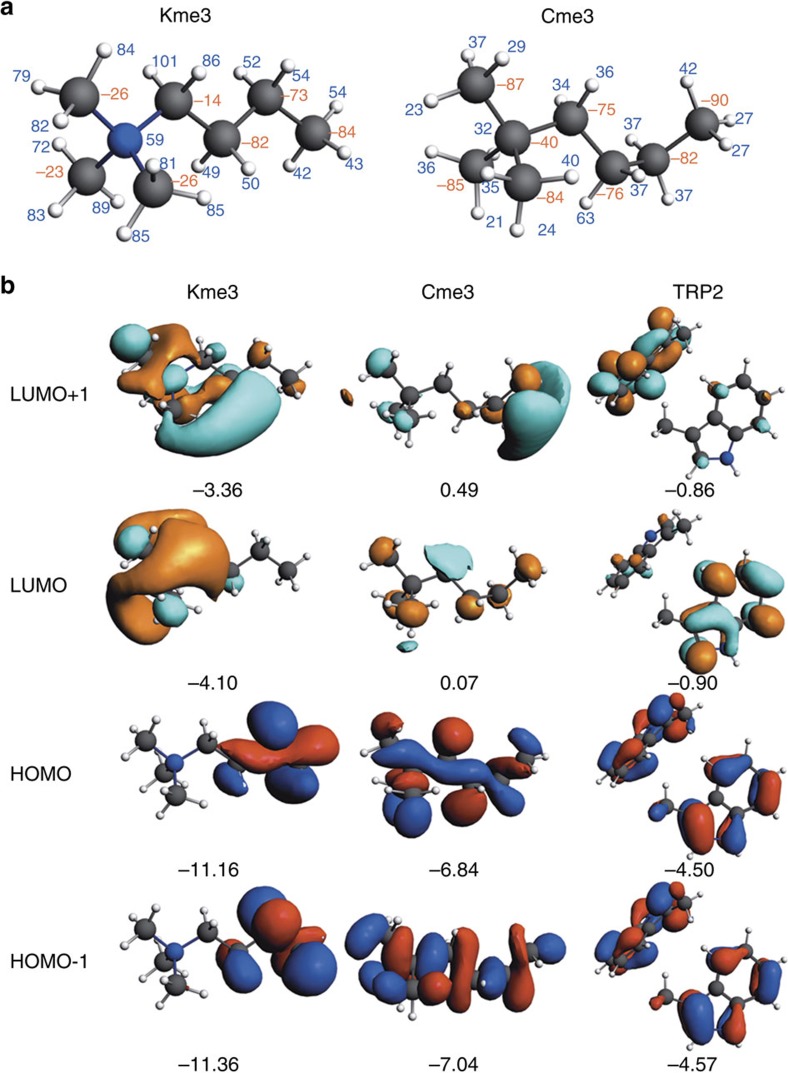
Computational analysis of TRP2–Kme3 and TRP2–Cme3 interactions. (**a**) VDD atomic charges (in mili-a.u.) of Kme3 and Cme3, computed at BLYP-D3BJ/TZ2P using X-ray structures of the full systems (red, negative; blue, positive); (**b**) Frontier orbitals (with orbital energies in eV) of Kme3, Cme3 and TRP2, computed at BLYP-D3BJ/TZ2P using X-ray structures of the full systems (isosurface drawn at 0.03).

The more attractive Δ*V*_elstat_ in case of Kme3 goes hand in hand with the significantly more positive charge on all atoms in the Kme3 ammonium, as inferred from our Voronoi deformation density (VDD) atomic charges^43^ (Fig. 3a). The nitrogen atom in Kme3 carries a positive charge of +59 mili-a.u., which has to be compared with the negative charge of −40 mili-a.u. on the structurally analogous carbon atom in the overall neutral Cme3. Importantly, the hydrogen atoms of the trimethylammonium group of Kme3 are also significantly more positively charged than the corresponding ones of the tert-butyl group in Cme3. For example, the hydrogen atom closest to the reader's TRP2 fragment has an atomic charge of +84 and +29 mili-a.u. in Kme3 and Cme3, respectively (Fig. 3a).

Our Kohn–Sham molecular orbital analyses show that the enhanced orbital interactions Δ*E*_oi_ in JARID1A–Kme3 result from both, stronger donor–acceptor orbital interactions and stronger polarization of the TRP2 fragment in the presence of the positively charged Kme3 than in the case of the neutral Cme3. Thus, the VDD analyses based on the two molecular fragments^43^ reveal a small but significant charge transfer of 0.04 electrons from the occupied *π* fragment molecular orbitals (FMOs) on TRP2 to virtual σ*_C–N_ and σ*_C–H_ type FMOs on Kme3 whereas essentially no charge is transferred to FMOs on Cme3. One reason is the much lower energy of the acceptor orbitals in the positively charged Kme3 (Fig. 3b). Another reason is the better overlap between TRP2 *π* orbitals and the acceptor orbitals of Kme3. This originates from the fact that the low-energy virtual orbitals of Kme3 are mainly localized on the positive trimethylammonium group through which Kme3 binds to TRP2, as can be seen in the realistic three-dimensional plots of relevant FMOs in Fig. 3b. The low-energy orbitals of Cme3 are more delocalized with less amplitude on the tert-butyl group close to TRP2. Consequently, in most cases TRP2–Kme3 overlaps are significantly larger than TRP2–Cme3 overlaps, as shown for the TRP2 highest occupied molecular orbital (HOMO) and HOMO-1 and the Kme3 or Cme3 lowest unoccupied molecular orbital (LUMO) and LUMO+1 (Supplementary Table 2).

### WaterMap calculations

**Figure 4:**
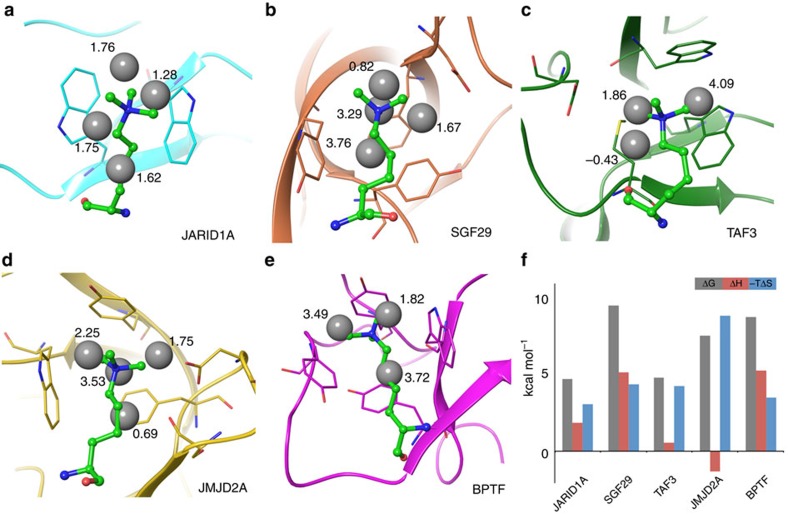
WaterMap calculations for the solvation of aromatic cages of reader proteins. (**a**) JARID1A; (**b**) SGF29; (**c**) TAF3; (**d**) JMJD2A; (**e**) BPTF. Superimposed Kme3 side chain and water molecules are presented as green stick and grey spheres. Numbers adjacent to grey spheres represent the value of the free energy (Δ*G*) for individual water molecule; (**f**) Thermodynamic parameters for the solvation of the aromatic cages of five reader proteins used in this study.

Next, we ran WaterMap calculations for all five systems to evaluate the contribution of aromatic cage desolvation to the affinity of Kme3 and Cme3 for reader proteins. WaterMap computes thermodynamic quantities (free energy, enthalpy and entropy) for simulated water molecules around a protein-binding site using explicit solvent molecular dynamics simulation and thermodynamic characterization. In short, regions of high solvent density from the molecular dynamic simulations are clustered into ‘hydration sites', and thermodynamic quantities for these sites are calculated using inhomogeneous solvation theory^44^,^45^. For all five reader proteins, two to four high-energy hydration sites were identified within the aromatic cage (Fig. 4a–e). These hydration sites are displaced from the aromatic cage by both the Kme3 and Cme3 side chain, but not by the H3G4 peptide. The total free energy contributed by desolvating the aromatic cage (determined as the difference in WaterMap scores between Kme3 and Gly) ranges from 4.3 kcal mol^−1^ for JARID1A to 8.7 kcal mol^−1^ for SGF29. Depending on the composition of the cage, this free energy reward can be both entropically and enthalpically driven (Fig. 4f and Supplementary Figs 4 and 5). For example, both TAF3 and JMJD2A contain an Asp residue that can form hydrogen bonds with the binding site water molecules, resulting in more favourable enthalpy of the hydration sites in the cage, hence more unfavourable change in enthalpy on displacing those waters on Kme3/Cme3 binding. On the other hand, the BPTF cage is completely surrounded by aromatic residues, producing an enthalpically unfavourable environment for water and therefore a favourable free energy change from water displacement on Kme3/Cme3 binding.

## Discussion

The advances of experimental and theoretical tools developed in the past decade have enabled more extensive analysis of the origins of some genuinely important biomolecular recognition phenomena, including the molecular basis of the hydrophobic effect(s) in protein–ligand interactions and the fundamentals of the receptor–neurotransmitter interactions in neurochemistry^27^,^46^. This study comprehensively examines the origin of the biomolecular recognition between naturally occurring trimethyllysine-containing histone proteins and their interacting reader proteins that are involved in epigenetic gene regulation processes. We use the physical–organic chemistry approaches, supported by high-resolution structural analyses of reader–histone interactions, to elucidate the molecular/chemical basis of one of the fundamental non-covalent interactions in epigenetics. Analyses of crystal and solution structures of free (unbound) reader proteins and reader–Kme3 complexes have illustrated that the reader's aromatic cage is largely preformed and does not undergo induced fit for binding of histone substrates (Supplementary Fig. 6). The predominantly static nature of the aromatic cage has an advantage over a more flexible recognition site because it minimizes the loss of conformational entropy of the protein on ligand binding^8^. Binding of the flexible and highly unstructured histone to reader proteins, however, results in a significant conformational change of the histone resulting in a more unfavourable entropy of binding for longer histone peptides relative to shorter histone counterparts^33^.

On the basis of the studies of the related proteins that possess the aromatic cages for the recognition of positively charged methylammonium groups, it has been suggested that epigenetic readers recognize Kme3 via cation–*π* interactions^16^,^27^,^37^,^47^. Our integrated thermodynamic, structural and computational studies clearly confirm the presence of favourable cation–*π* interactions in the readout of H3K4me3 by reader domains of JARID1A, TAF3, BPTF and JMJD2A. Previous examination of the recognition of neutral Cme3 by HP1 chromodomain, a reader of H3K9me3 that contains an aromatic cage comprising two tyrosine and one tryptophan residues, revealed that HP1 binds to H3C9me3 with substantially lower affinity than H3K9me3, thus suggesting that the positive charge of Kme3 is crucial for the association of HP1–H3K9me3 (ref. 16). Comprehensive structural data on JARID1A, TAF3 and SGF29 in complex with H3C4me3, as described in this work, provide clear evidence that the Cme3 side chain is well positioned inside the aromatic cages of these three reader proteins in the same manner as the positively charged Kme3 (Fig. 2) and thus enable us to interpret the binding calorimetric data (Table 1). Out of three possible mechanisms (that should always be considered in the interpretation of any protein–ligand system), that is, solute–solute interactions, desolvation of ligand (in this case Kme3) and desolvation of protein (in this case aromatic cage), that govern the recognition of Kme3 by reader proteins, we can exclude desolvation of the Kme3 side chain, because charged residues are highly soluble in aqueous media and have to pay a big desolvation penalty to become desolvated. In this regard, it is essential that the energetically unfavourable desolvation of Kme3 is fully compensated (or more correctly overcompensated) by energetically favourable protein–ligand interactions and protein desolvation to provide a strong binding force for the specific recognition of Kme3 by reader proteins. Based on ITC experiments, our observed enthalpy-driven association of positively charged Kme3 (relative to Cme3) to the electron-rich aromatic cage of several reader proteins has its molecular origin in strong cation–*π* interactions. In addition, the methylene groups of the side chain of Kme3 located within van der Waals distance of the aromatic cages, contribute to the overall binding affinity via weaker, but still favourable, CH–*π* interactions^38^,^39^. Our quantum mechanical studies, furthermore, reveal that reader–Kme3 association has the strongest dispersion contribution (similar to reader–Cme3), but that the differences in binding affinities between Kme3 and Cme3 are primarily a result of disparities in electrostatic interactions and orbital interactions (Table 3).

Despite the universally recognized phenomenon that biomolecular processes take place in aqueous media and that the hydrophobic effect is a primary determinant of biomolecular association, the role of explicit water molecules has often been ignored in analyses of biomolecular recognition events^48^,^49^, although recent advances have enabled more detailed analysis of the role of water molecules in binding^46^,^50^,^51^. Energetically favourable desolvation of protein-binding sites, however, often determines the magnitude of protein–ligand association^52^,^53^. Our observations that binding affinities of H3G4 with JARID1A and TAF3 are drastically reduced when compared with H3K4me3 led to the hypothesis that the aromatic cages are occupied by high-energy water molecules. Although difficult to confirm experimentally, WaterMap calculations performed on five representative reader proteins (both in apo and holo forms) provided evidence that water molecules located inside the aromatic cages exhibit significant unfavourable free energy (Fig. 4 and Supplementary Table 3). These high-energy water molecules are displaced by Kme3 side chain on binding, which consequently provide a substantial favourable contribution to Kme3 binding.

Collectively, the experimental and computational work presented here suggests that the association between trimethyllysine-containing histones and epigenetic reader domain proteins is driven by favourable cation–*π* interactions and the favourable release of high-energy structured water molecules that occupy the aromatic cages of reader proteins. Our study highlights the hitherto neglected, yet essential contribution of water in a molecular readout process in the established area of epigenetics. This study, furthermore, sheds light on the design of small molecule probes that specifically recognize readers of trimethyllysine. In comparison with the advances in development of inhibitors of other epigenetic targets, including bromodomains and various eraser/writer enzymes, there has been very limited success in identification of probes for readers of Kme3 (refs 54, 55). Towards this aim, our study provides valuable experimental and computational data needed for the medicinal chemistry community to design and develop potent and selective small molecule inhibitors with therapeutic potential.

## Methods

### General experimental procedures

All experiments were conducted under the following conditions, unless stated otherwise. Commercially available compounds were supplied by commercial sources and used without any further purification. Dry solvents were obtained by purification of HPLC grade solvents over activated alumina column using an MBraun SPS800 solvent purification system. When stated, degassing of solvents was performed for each reaction individually by passing through N_2_ (g) for a period of at least 30 min before use. Compound purification done by column chromatography, was carried out using Silica gel, MerckTM grade (pore size 60 Å; particle size 230–400 mesh, 40–63 μm). Reaction progress was monitored by glass thin-layer chromatography plates (TLC Silica gel 60G, F254, Merck, Germany) and observed by ultraviolet light and/or by staining in ninhydrin or permanganate. Compound analyses done by ^1^H NMR, were recorded on a Varian Inova 400 at 400 MHz. ^13^C NMR data were either recorded using a Bruker Avance III 500 MHz at 125 MHz or a Varian Inova 400 at 101 MHz. Reported chemical shifts are in p.p.m., moving from high to low frequency and referenced to the residual solvent resonance. Reported coupling constants (*J*) are noted in hertz (Hz). To assign multiplicity of signals the following standard abbreviations were used: s, singlet; d, doublet; t, triplet; q, quartet; quint, quintet; m, multiplet; and br, broad. When possible, ^1^H assignments were made using appropriate two-dimensional NMR methods, such as correlation spectroscopy, heteronuclear single-quantum correlation spectroscopy and heteronuclear multiple-bond correlation spectroscopy. Mass spectrometry and chromatography analysis were done using a Shimadzu UFLC LC-20AD liquid chromatography/mass spectrometry system, equipped with a RPC18 200 × 2 guard column. Typical conditions for a run are: 157 bar, mobile phase; 2 min 5% MeCN 95% H_2_O, in 16 min decreasing polarity to 100% MeCN, 5 min of 100% MeCN, in 2 min increasing polarity to 95% H_2_O for 5 min. Ultraviolet/visible detection of this machine was done by Ultraviolet Visible Shimadzu SPD-M20A (200–600 nm), while mass spectrometry analyses was done using the Thermo scientific LCQ Fleet. HPLC trace analyses were done on a Shimadzu liquid chromatography system; DGU 20A5, using a SPD 20A ultraviolet detector at 214 nm. The machine is equipped with a Gemini-NX 3 C18 column. Typical conditions for a run are: 1 min at 5% MeCN in 95% H_2_O (with 0.1% trifluoroacetic acid (TFA)), increase over 30 min to 100%, keep this for 5 min, then over 5 min the concentration is decreased to 5% MeCN in 95% H_2_O (with 0.1% TFA).

### Synthesis of Fmoc-L-Cme3

Supplementary Fig. 7 shows the schematic presentation of the synthetic protocol for the preparation of Fmoc-L-Cme3 (**6**).

### Synthesis of (1)

Boc-Asp(OH)-OtBu (5.81 g, 20 mmol, 1 equivalent), 4-dimethylaminopyridine (223.8 mg, 2 mmol, 0.1 equivalents) and *N*,*N*'-dicyclohexylcarbodiimide (4.95 g, 24 mmol, 1.2 equivalents) were dissolved in dry CH_2_Cl_2_ (40 ml) under N_2_ atmosphere. To this solution was added ethanethiol (4.7 ml, 64 mmol 3.2 equivalents). After 4 h of stirring the solvent was removed under reduced pressure. The crude product was purified by column chromatography (SiO_2_, EtOAc in *n*-pentane 5–20%). This yielded thioester **1** (6.26 g, 18.8 mmol, 94%) as a pale yellow oil: [α]^25^_D_ +43.4 (c 1.00, CH_3_Cl). FT-IR v_max_ (cm^−1^): 3,436, 2,980, 2,932, 1,715, 1,688, 1,495, 1,367, 1,250, 1,150, 1,059, 1,023 and 847. ^1^H NMR (400 MHz, CDCl_3_) δ: 5.42 (d, *J*=8.0 Hz, 1H, NH), 4.48–4.36 (m, 1H, αCH), 3.10 (dq, *J*=17.0, 5.0 Hz, 2H, βCH_2_), 2.96–2.78 (m, 2H, SCH_2_), 1.46 (s, 9H, C(CH_3_)_3_), 1.44 (s, 9H, C(CH_3_)_3_) and 1.29–1.22 (m, 3H, CH_3_). ^13^C NMR (101 MHz, CDCl_3_) δ: 196.9, 169.6, 155.2, 82.3, 79.7, 50.8, 45.5, 28.2, 27.8, 23.4 and 14.6. HRMS, calculated for C_15_H_27_NO_5_SNa [M+Na]^+^ 356.1508, found 356.1511.

### Synthesis of (2)

To a suspension of Pd/C (375 mg, 10% Pd on activated carbon, 6 wt%) and thioester **1** (6.26 g, 18.8 mmol, 1 equivalent) in degassed dry CH_2_Cl_2_ (40 ml) was added triethylsilane (9 ml, 56.3 mmol, 3 equivalents). The solution was stirred for 90 min, while cooling on a water bath. The black suspension was filtered through celite, concentrated and purified by column chromatography (SiO_2_, EtOAc in *n*-heptane 5–25%). This eventually yielded aldehyde **2** (4.84 g, 17.7 mmol, 95%) as a clear colourless oil, which solidified over time: [α]^25^_D_ −24.2 (c 1.50, EtOH). FT-IR v_max_ (cm^−1^): 3,370, 2,980, 2,935, 1,714, 1,501, 1,368, 1,251, 1,151, 1,054 and 847. ^1^H NMR (400 MHz, CDCl_3_) δ: 9.74 (s, 1H, C(O)H), 5.34 (d, *J*=7.5 Hz, 1H, NH), 4.58–4.39 (m, 1H, αCH), 2.98 (qd, *J*=18.0, 5.0 Hz, 2H, βCH_2_), 1.47 (s, 9H, C(CH_3_)_3_) and 1.44 (s, 9H, C(CH_3_)_3_). ^13^C NMR (101 MHz, CDCl_3_) δ: 199.3, 169.8, 155.3, 82.4, 79.8, 49.2, 46.1, 28.2 and 27.7. HRMS, calculated for C_13_H_23_NO_5_Na [M+Na]^+^ 296.1474, found 296.1471.

### Synthesis of (3)

To a suspension of methyltriphenylphosphonium bromide (2.23 g, 6.16 mmol 1.1 equivalents) in dry tetrahydrofuran (THF; 30 ml) under N_2_ atmosphere, was added NaHMDS (3.1 ml, 6.16 mmol, 2.0 M in THF, 1.1 equivalents). Aldehyde **2** (1.08 g, 3.66 mmol, 1 equivalent) was dissolved in dry THF (15 ml) and added to the solution after 30 min of stirring. Subsequently, the reaction mixture was stirred for 20 h and then quenched by the addition of KHSO_4 (aq)_ (60 ml, 1 M). The aqueous layer was extracted with EtOAc (3 × 25 ml) and the combined organic extracts were washed with H_2_O (50 ml) and brine (50 ml). The organic layer was dried over Na_2_SO_4_, filtered and evaporated under vacuum. The crude product was purified by silica column chromatography (SiO_2_, EtOAc in *n*-heptane 5–20%), affording **3** (919 mg, 3.385 mmol, 60%) as a clear colourless oil. [α]^25^_D_ +10.3 (c 0.84, MeOH). FT-IR v_max_ (cm^−1^): 3,352, 2,980, 2,933, 1,715, 1,496, 1,367, 1,251, 1,154, 918 and 847. ^1^H NMR (400 MHz, CDCl_3_) δ: 5.79–5.63 (m, 1H, CH_2_=CH), 5.16–5.09 (m, 2H, CH_2_=CH), 5.05 (d, *J*=7.5 Hz, 1H, NH), 4.25 (dd, *J*=19.0 Hz, 8.5 Hz 1H, αCH), 2.63–2.39 (m, 2H, βCH_2_), 1.46 (s, 9H, C(CH_3_)_3_) and 1.44 (s, 9H, C(CH_3_)_3_). ^13^C NMR (101 MHz, CDCl_3_) δ: 171.1, 155.2, 132.5, 118.7, 81.9, 79.6, 53.3, 37.0, 28.3 and 28.0. HRMS, calculated for C_14_H_25_NO_4_Na [M+Na]^+^ 294.1681, found 294.1683.

### Synthesis of (4)

To a solution of **3** (918 mg, 3.39 mmol, 1 equivalent) in dry CH_2_Cl_2_ (30 ml) under N_2_ atmosphere, were added second generation Grubbs catalyst (434 mg, 0.51 mmol, 0.15 equivalents) and 4,4-dimethyl-1-pentene (1,860 μl, 15.54 mmol, 4 equivalents). This solution was stirred for 24 h at 50 °C. After cooling down, the solvent was evaporated under reduced pressure. The crude product was purified by column chromatography (SiO_2_, EtOAc in *n*-heptane 0–10%), affording **4** (650 mg, 1.9 mmol, 56%). [α]^25^_D_ −5.4 (c 0.93, MeOH). FT-IR v_max_ (cm^−1^): 3,337, 2,954, 1,716, 1,495, 1,365, 1,248, 1,153, 970 and 847. ^1^H NMR (400 MHz, CDCl_3_) (Z: E ratio 1: 4.7, most abundant isomer) δ: 5.49–5.58 (m, 1H, CH=CH), 5.32–5.20 (m, 1H, CH=CH), 5.01 (d, *J*=8.0 Hz, 1H, NH), 4.29–4.16 (m, 1H, αCH), 2.54–2.33 (m, 2H, βCH_2_), 1.88 (dd, *J*=7.5, 1.0 Hz, 2H, ɛCH_2_), 1.47 (s, 9H, C(CH_3_)_3_), 1.44 (s, 9H, C(CH_3_)_3_) and 0.87 (s, 9H, C(CH_3_)_3_). ^13^C NMR (126 MHz, CDCl_3_) δ: 171.4, 155.1, 132.2, 125.8, 81.7, 79.5, 53.6, 47.1, 35.8, 30.8, 29.3, 28.3 and 28.1. HRMS, calculated for C_19_H_35_NO_4_Na [M+Na]^+^ 364.2464, found 364.2478.

### Synthesis of (5)

To a suspension of Pd/C (140 mg, 10% Pd on activated carbon, 25 wt%) in dry CH_2_Cl_2_ (20 ml), was added **4** (558 mg, 1.63 mmol, 1 equivalent). The solution was vigorously stirred under H_2_ atmosphere for 24 h. The black suspension was filtered through celite and washed with CH_2_Cl_2_ (3 × 25 ml). The filtrate was concentrated under reduced pressure yielding **5** (530 mg, 1.54 mmol, 95%) as a slightly brown oil. [α]^25^_D_ −14.0 (c 1.00, MeOH). FT-IR v_max_ (cm^−1^): 3,350, 2,954, 2,865, 1,770, 1,498, 1,392, 1,366, 1,249, 1,154 and 849. ^1^H NMR (500 MHz, CDCl_3_) δ: 4.92 (d, *J*=8.0 Hz, 1H, NH), 4.09 (dd, *J*=13.0, 7.0 Hz, 1H, αCH), 1.60–1.48 (m, 2H, βCH_2_), 1.40 (s, 9H, C(CH_3_)_3_), 1.37 (s, 9H, C(CH_3_)_3_), 1.30–1.13 (m, 4H, γCH_2_ and δCH_2_), 1.12–1.04 (m, 2H, ɛCH_2_) and 0.79 (s, 9H, C(CH_3_)_3_). ^13^C NMR (126 MHz, CDCl_3_) δ: 172.2, 155.4, 81.6, 79.5, 54.0, 44.0, 33.0, 30.3, 29.4, 28.4, 28.0, 26.1 and 24.3. HRMS, calculated for C_19_H_37_NO_4_Na [M+Na]^+^ 366.2620, found 366.2619.

### Synthesis of (6)

Protected **5** (295 mg, 0.86 mmol, 1 equivalent) was dissolved in a mixture of TFA: dichloromethane (30 ml, 2:1) and left stirring for 5 h. The solvent was removed under vacuum and the resulting crude product was redissolved in H_2_O: dioxane (30 ml, 1:1) and the pH of the solution was adjusted to pH 8–9 by the addition of NaHCO_3_. Subsequently, Fmoc-OSu (435 mg, 1.29 mmol, 1.5 equivalents) was added to the solution. After stirring for 16 h the solution was acidified to pH 3 by addition of HCl _(aq)_ (1 M) and extracted with EtOAc (5 × 20 ml). The combined organic extracts were washed with brine (50 ml), dried over Na_2_SO_4_, filtered and concentrated under reduced pressure. The crude oil was purified by column chromatography (SiO_2_, MeOH in CH_2_Cl_2_ and a few drops of AcOH, 1–4%), affording **6** (295 mg, 0.72 mmol, 84%) as a clear viscous oil. [α]^25^_D_ −2.5 (c 0.16, MeOH). FT-IR v_max_ (cm^−1^): 3,326, 2,952, 2,862, 1,710, 1,520, 1,451, 1,214, 1,079, 758 and 739. ^1^H NMR (400 MHz, CDCl_3_) δ: 7.77 (d, *J*=6.0 Hz, 2H, 2 × ArCH), 7.64–7.50 (m, 2H, 2 × ArCH), 7.40 (t, *J*=7.4 Hz, 2H, 2 × ArCH), 7.31 (t, *J*=7.0, 2H, 2 × ArCH), 5.37–5.20 (m, 1H, NH), 4.54–4.33 (m, 3H, αCH and OCH_2_), 4.28–4.17 (m, 1H, CH), 1.97–1.82 (m, 1H, βCH), 1.78–1.66 (m, 1H, βCH), 1.44–1.10 (m, 6H, γCH_2_ and δCH_2_ and ɛCH_2_) and 0.86 (s, 9H, C(CH_3_)_3_). ^13^C NMR (126 MHz, CDCl_3_) δ: 177.5, 156.1, 143.9, 141.3, 127.7, 127.1, 124.9, 120.0, 67.1, 53.9, 47.2, 43.9, 32.4, 30.3, 29.4, 26.1 and 24.2. HRMS, calculated for C_25_H_31_NO_4_Na [M+Na]^+^ 432.2151, found 432.2153.

### Solid-phase peptide synthesis

Ten mer histone peptides were synthesized by solid-phase peptide synthesis applying Fmoc chemistry. Peptides contain a carboxylic acid at the C terminus and were made on Wang resin and couplings were done in dimethylformamide (DMF) with Fmoc-protected amino acid (3.0 equivalents), diisopropylcarbodiimide (3.3 equiv.) and hydroxybenzotriazole (3.6 equivalents). Completion of the reaction was determined with the Kaiser test, and removal of Fmoc was achieved by treatment with a large excess of piperidine (20%) in DMF for 20–30 min. Every wash step was performed with 3 × DMF and after building completion the Fmoc was removed followed by wash 3 × DMF and 3 × Et_2_O continued by drying of the resin in vacuo. The peptides were cleaved from the resin by a mixture of TFA (92.5%), H_2_O (2.5%), tri-isopropylsilane (2.5%) and ethane-1,2-dithiol (2.5%). After mixing and shaking for 4–5 h, the product peptide was precipitated in Et_2_O, and the Et_2_O was decanted after centrifugation (3,500 r.p.m., 3 min, Hermle 220.72 v04). Histone peptides were analysed by liquid chromatography–mass spectrometry and purified by preparative HPLC (Supplementary Figs 8–13). Purified histone peptides were analysed by ^19^F NMR spectroscopy, which provided evidence that they appear as TFA salts.

### Preparation and purification of reader proteins

Reader proteins were prepared and purified following the previously reported procedure^33^. Briefly, the reader domains of BPTF, JMJD2A, JARID1A, TAF3 and SGF29 were expressed in *Escherichia coli* Rosetta BL21 DE3 pLysS hosts, using Terrific Broth medium. The bacteria were cultured to OD600 ∼0.6 at 37 °C after which they were induced with 0.5 mM isopropyl-b-D-thiogalactoside overnight at 16 °C. Proteins were purified using Ni-NTA beads for 6xHis-tagged proteins or glutathione sepharose beads for GST tagged proteins, respectively. After purification, the 6xHis tag was cleaved from JMJD2A and SGF29 using TEV-protease and the GST tag was cleaved from TAF3 using thrombin. Protein were purified by size-exclusion chromatography using a Superdex 75 column (GE Healthcare). SGF29 was eluted in 25 mM Tris, 50 mM NaCl, 1 mM dithiothreitol at pH 7.5; JMJD2A and TAF3 were eluted in 50 mM Tris at pH 7.5; BPTF and JARID1A were eluted in 50 mM Tris, 20 mM NaCl at pH 7.5. All proteins were made filter sterile and stored at 4 °C until further use.

### Isothermal titration calorimetry

Concentrations of histone peptides were measured by ultraviolet spectroscopy at 205 nm, following the previously reported method^56^. All histone peptides were titrated to the same batch of reader proteins. Generally, 350–600 μM of H3K4me3 or H3C4me3 peptides were titrated to 25–40 μM of protein, except for JMJD2A–H3C4me3 (200 μM JMJD2A, 3 mM H3C4me3). H3G4 (5 mM) was titrated to JARID1A (330 μM) and H3G4 (3 mM) was titrated to TAF3 (200 μM). Each ITC titration consisted of 19 injections. ITC experiments were performed on the fully automated Microcal Auto-iTC200 (GE Healthcare Life Sciences, USA). Heats of dilution for histone peptides were determined in control experiments, and were subtracted from the titration binding data before curve fitting. Curve fitting was performed by Origin 6.0 (Microcal Inc., USA) using one set of sites binding model. For each reader–histone system, 5–7 independent ITC experiments were carried out. Measurements of heat capacities were typically done in the interval of 10–30 °C, in triplicate at each temperature.

### X-ray crystallography

The tandem tudor domain of human SGF29 (residues 115–293) was cloned into a pET-28a-MHL vector, and is expressed, purified as described before^22^. The purified SGF29 is concentrated to 20 mg ml^−1^ as a stock and frozen at −80 °C for future use. Purified SGF29 (15 mg ml^−1^) was mixed with histone peptide H3C4me3 in a molecular ratio of 1:3, and the complex was crystallized in a buffer containing 0.1 M Bis-Tris, pH 5.5, 27% PEG3350, 200 mM ammonium sulphate and 5 mM strontium chloride. Before flash-frozen in liquid nitrogen, the crystals were soaked in a cryoprotectant buffer containing 88% reservoir solution and 12% glycerol.

Human JARID1A PHD finger (aa 330–380) was PCR amplified, and cloned into a modified pET28b vector (Novagen) with an N-terminal 10xHis-SUMO tandem tag. JARID1A PHD finger used for crystallization was expressed in the *E. coli* BL21 (Novagen) induced overnight by 0.2 mM isopropyl β-D-thiogalactoside at 25 °C in the LB medium supplemented with 0.1 mM ZnCl_2_. The collected cells were suspended in 500 mM NaCl, 20 mM Tris, pH 8.5. After cell lysis and centrifugation, the supernatant was applied to a HisTrap (GE Healthcare) column and the protein was eluted with a linear imidazole gradient from 20 mM to 500 mM, followed by tag cleavage using ULP1. A HisTrap column was used to remove the cleaved 10xHis-SUMO tag after removal of imidazole by desalting. The JARID1A PHD sample flow-through was then pooled, concentrated and polished by size-exclusion chromatography on a Superdex 75 16/60 column (GE Healthcare) under the elution buffer: 150 mM NaCl, 20 mM Tris, pH 8.5. The resultant peak of JARID1A PHD finger was then concentrated to ∼17 mg ml^−1^, split into small aliquots and frozen in liquid nitrogen for future use.

As for human TAF3, the PHD finger construct 885–915 was cloned, expressed and purified using essentially the same strategy as JARID1A PHD finger. TAF3 PHD finger was concentrated to ∼25 mg ml^−1^ and aliquoted for future use.

Crystallization was performed via the sitting drop vapour diffusion method under 4 °C by mixing equal volume (0.2–1.0 μl) of JARID1A PHD-H3C4me3 complex (1:1.8 molar ratio, 14–16 mg ml^−1^) and reservoir solution containing 0.02 M sodium-l-glutamate, 0.02 M DL-alanine, 0.02 M glycine, 0.02 M DL-lysine HCl, 0.02 M DL-serine, 0.1 M Tris, 0.1 M Bicine, pH 8.5, 12.5% MPD, 12.5% PEG 1 K, 12.5% PEG3350. As for TAF3 PHD-H3C4me3 complex (1:1.4 molar ratio, 22–24 mg ml^−1^), the crystal was grown in the reservoir solution containing 0.03 M magnesium chloride, 0.03 M calcium chloride, 0.1 M MES, 0.1 M imidazole, pH 6.5, 15% PEGMME 550, 15% PEG 20 K. The complex crystals were directly flash-frozen in liquid nitrogen with reservoir solution as cryoprotectant for data collection. The diffraction data were collected at the beamline BL17U of the Shanghai Synchrotron Radiation Facility at 0.9793 Å. All diffraction images were indexed, integrated and merged using HKL2000 (ref. 57). The structure was determined by molecular replacement using MOLREP^58^ with the free JARID1A PHD finger (PDB ID: 2KGG) and free TAF3 PHD finger (PDB ID: 2K16) as the search model. Structural refinement was carried out using PHENIX^59^, and iterative model building was performed with COOT^60^. Detailed data collection and refinement statistics are summarized in Table 2. Structural figures were created using the PYMOL (http://www.pymol.org/) program.

### Quantum-chemical analyses

All calculations for TRP2-Kme3 and TRP2-Cme3 complexes were carried out with the ADF program using dispersion-corrected density functional theory at the BLYP-D3BJ/TZ2P level of theory^40^,^41^. The effect of solvation was simulated by means of the Conductor like Screening Model (COSMO) of solvation as implemented in ADF. The approach has been benchmarked against highly correlated post-Hartree–Fock methods and experimental data and was found to work reliably^61^,^62^,^63^.

The bonding mechanism in our model complexes have been further analysed using quantitative (Kohn–Sham) molecular orbital theory in combination with an EDA^42^,^64^. The bond energy in aqueous solution Δ*E*(aq) consists of two major components, namely, the strain energy Δ*E*_strain_(aq) associated with deforming the Kme3 (or Cme3) and the reader from their own equilibrium structure to the geometry they adopt in the complex, plus the interaction energy Δ*E*_int_(aq) between these deformed solutes in the complex (see equation (1)):


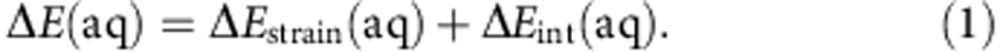


To arrive at an understanding of the importance of desolvation phenomena during the complexation process, we separate the interaction energy Δ*E*_int_(aq) into the effect caused by the change in solvation Δ*E*(desolv) and the remaining intrinsic solute-solute interaction Δ*E*_int_ between the unsolvated fragments in vacuum:


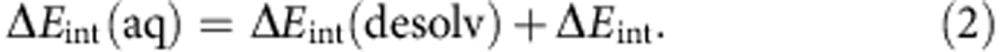


In the EDA, the intrinsic interaction energy Δ*E*_int_ can be further decomposed as shown in equation (3):


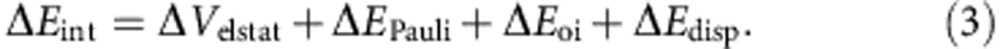


Here Δ*V*_elstat_ corresponds to the classical electrostatic interaction between the unperturbed charge distributions of the deformed fragments that is usually attractive. The Pauli repulsion Δ*E*_Pauli_ comprises the destabilizing interactions between occupied orbitals and is responsible for the steric repulsions. The orbital interaction Δ*E*_oi_ accounts for charge transfer (donor–acceptor interactions between occupied orbitals on one moiety with unoccupied orbitals of the other, including the HOMO–LUMO interactions) and polarization (empty/occupied orbital mixing on one fragment due to the presence of another fragment). Finally, the Δ*E*_disp_ term accounts for the dispersion interactions based on Grimme's DFT-D3BJ correction. Furthermore, the charge distribution has been analysed using the VDD method^43^.

### WaterMap calculations

WaterMap has been described in detail in previous works^52^,^65^. All calculations were run in with default settings. In brief, a 2 ns molecular dynamic simulation of the reader proteins with the peptide removed, is performed using the Desmond molecular dynamic engine^66^,^67^ with the OPLS2.1 force field^68^,^69^. Protein atoms are constrained throughout the simulation. Water molecules from the simulation are then clustered into distinct hydration sites. Enthalpy values for each hydration site are obtained by averaging over the non-bonded interaction for each water molecule in the cluster. Entropy values are calculated using a numerical integration of a local expansion of the entropy in terms of spatial and orientational correlation functions^44^,^45^. The contribution of water-free energy to the binding free energy of the peptide is approximated by the sum of the free energies of hydration sites displaced by the ligand on binding.

## Additional information

**Accession codes:** Coordinates of JARID1A PHD–H3(1-10)C4me3, TAF3 PHD–H3(1-10)C4me3 and SGF29 tandem tudor–H3(1-10)C4me3 complexes have been deposited into Protein Data Bank under accession codes 5C11, 5C13 and 5C0M, respectively.

**How to cite this article:** Kamps, J. A. G. *et al*. Chemical basis for the recognition of trimethyllysine by epigenetic reader proteins. *Nat. Commun.* 6:8911 doi: 10.1038/ncomms9911 (2015).

## Supplementary Material

Supplementary Information Supplementary Figures 1-13 and Supplementary Tables 1-3

## Figures and Tables

**Table 1:** Thermodynamic parameters for the associations of 10-mer H3K4me3 and H3C4me3 peptides (ART(Kme3/Cme3)QTARKS) to five reader proteins.

	H3K4me3				H3C4me3			
	*K*_d_ (μM)	Δ*G*° (kcal mol^−1^)	Δ*H*° (kcal mol^−1^)	-TΔ*S*° (kcal mol^−1^)	*K*_d_ (μM)	Δ*G*° (kcal mol^−1^)	Δ*H*° (kcal mol^−1^)	−TΔ*S*° (kcal mol^−1^)
JARID1A	0.094	−9.6±0.1	−11.0±0.1	1.4±0.1	0.34	−8.8±0.1	−7.4±0.1	−1.4±0.1
TAF3	0.024	−10.4±0.1	−10.9±0.1	0.5±0.2	0.79	−8.3±0.1	−5.2±0.1	−3.1±0.2
BPTF	0.49	−8.6±0.1	−13.1±0.1	4.5±0.1	0.76	−8.3±0.1	−10.0±0.2	1.7±0.2
SGF29	1.7	−7.9±0.1	−7.7±0.1	−0.2±0.1	1.4	−8.0±0.1	−7.9±0.1	−0.1±0.1
JMJD2A	0.94	−8.2±0.1	−13.1±0.2	4.9±0.2	16	−6.5±0.1	−8.1±0.1	1.6±0.1

^*^Values obtained from 5–7 repeated ITC experiments. The stoichiometry (histone peptide:reader protein, *n*)=0.95–1.05.

**Table 2:** Data collection and refinement statistics.

	JARID1A–H3C4me3	TAF3–H3C4me3	SGF29–H3C4me3
*Data collection*			
Space group	I432	P2_1_	P2_1_2_1_2_1_
			
Cell dimensions			
*a, b, c (Å)*	108.9, 108.9, 108.9	30.2, 50.1, 85.9	50.1,65.2,105.2
*α*, *β*, *γ* (°)	90, 90, 90	90, 90, 90	90, 90, 90
Resolution (Å)	50–2.8 (2.87–2.80)*	50–2.1 (2.14–2.10)	37.2–1.60 (1.63–1.60)
*R*_merge_	6.5 (79.8)	12.3 (66.9)	7.3 (77.5)
*I I σI*	64.9 (3.3)	17.1 (2.8)	17.6 (2.6)
Completeness (%)	99.5 (100)	99.6 (100)	99.1 (92.1)
Redundancy	17.1 (14.4)	3.7 (3.7)	6.8 (6.6)
			
*Refinement*			
Resolution (Å)	50–2.8	32.6–2.1	37.2–1.60
No. reflections	2,920	15,001	44,342
*R*_work_/*R*_free_	24.6/27.9	22.2/28.0	20.4/23.9
No. atoms			
Protein	399	2,064	2,865
Ligand/ion	56/2	228/8	84/10
Water	0†	78	381
*B*-factors			
Protein	97.3	34.9	16.8
Ligand/ion	80.3/86.9	29.2/25.8	19.7/34.6
Water		37.7	25.9
R.m.s. deviations			
Bond lengths (Å)	0.003	0.014	0.009
Bond angles (°)	0.698	1.58	1.384

^*^Values in parentheses are for highest-resolution shell.

^†^No water molecules are modelled due to high *B*-factor of the complex structure.

**Table 3:** Quantum-chemical bonding analysis (energies in kcal mol^−1^, distances in Å) in TRP2–Kme3 and TRP2–Cme3 systems in aqueous solution.

	TRP2–Kme3	TRP2–Cme3
Δ*E*(aq)	−10.2	−8.4
Δ*E*(aq)_strain_	0.1	0.3
Δ*E*(aq)_int_	−10.3	−8.7
Δ*E*(desolv)_int_	+17.3	+2.2
Δ*E*_int_	−27.6	−10.9
Δ*E*_Pauli_	20.8	14.1
Δ*V*_elstat_	−15.0	−5.4
Δ*E*_oi_	−13.0	−3.6
Δ*E*_disp_	−20.4	−16.0
d(H_Me_-C_TRP-6MR_)	3.38	3.15
d(H_Me_-C_TRP-5MR_)	2.78	3.16

^*^Computed at BLYP-D3BJ/TZ2P with COSMO to simulate aqueous solution. Structural rigidity imposed by the protein backbone is simulated through constraint geometry optimizations. See also equations (1)–(3) in Methods section.
