# Supplementary material for: Chemical basis for the recognition of trimethyllysine by epigenetic reader proteins
Source: Nat Commun. 2015 Nov 18;6:8911. doi: 10.1038/ncomms9911 (PMC4673829; doi:10.1038/ncomms9911)
Supplement: Supplementary Information — Supplementary Figures 1-13 and Supplementary Tables 1-3 [file ncomms9911-s1.pdf]

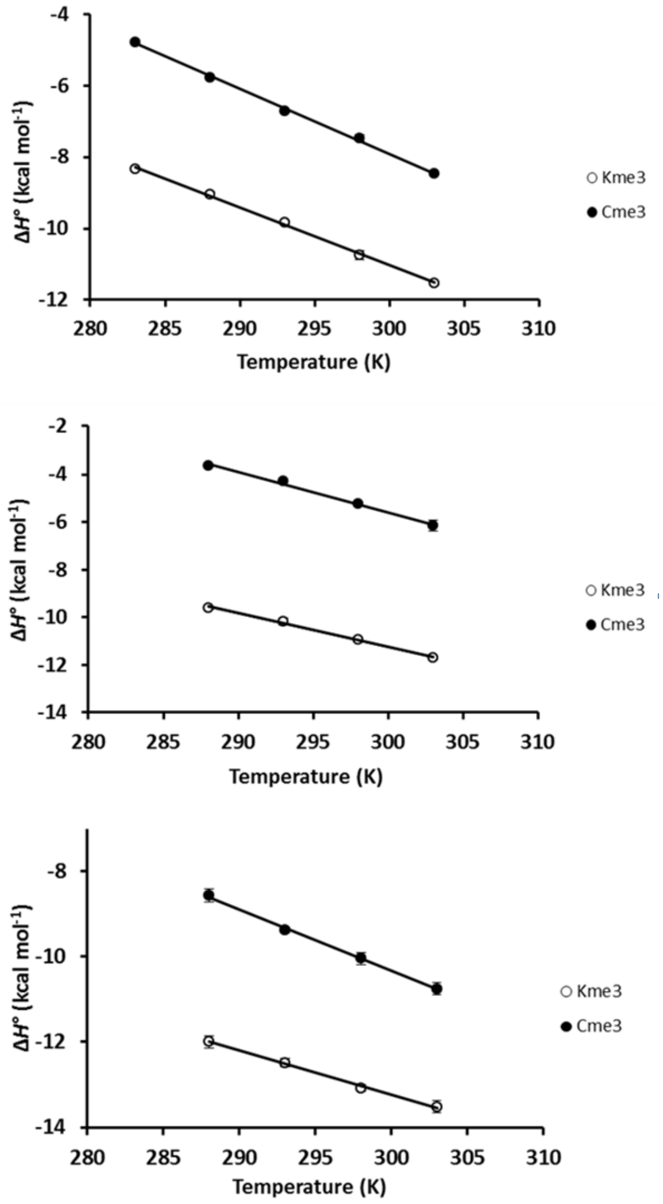

Supplementary Figure 1. Observed enthalpies of binding as a function of temperature between 283 K (or 288 K) and 303 K. Enthalpy of binding was measured by ITC with three independent titrations at each temperature. (**a**) JARID1A with H3K4me3 and H3C4me3, respectively; (**b**) TAF3 with H3K4me3 and H3C4me3, respectively; (**c**) BPTF with H3K4me3 and H3C4me3, respectively.

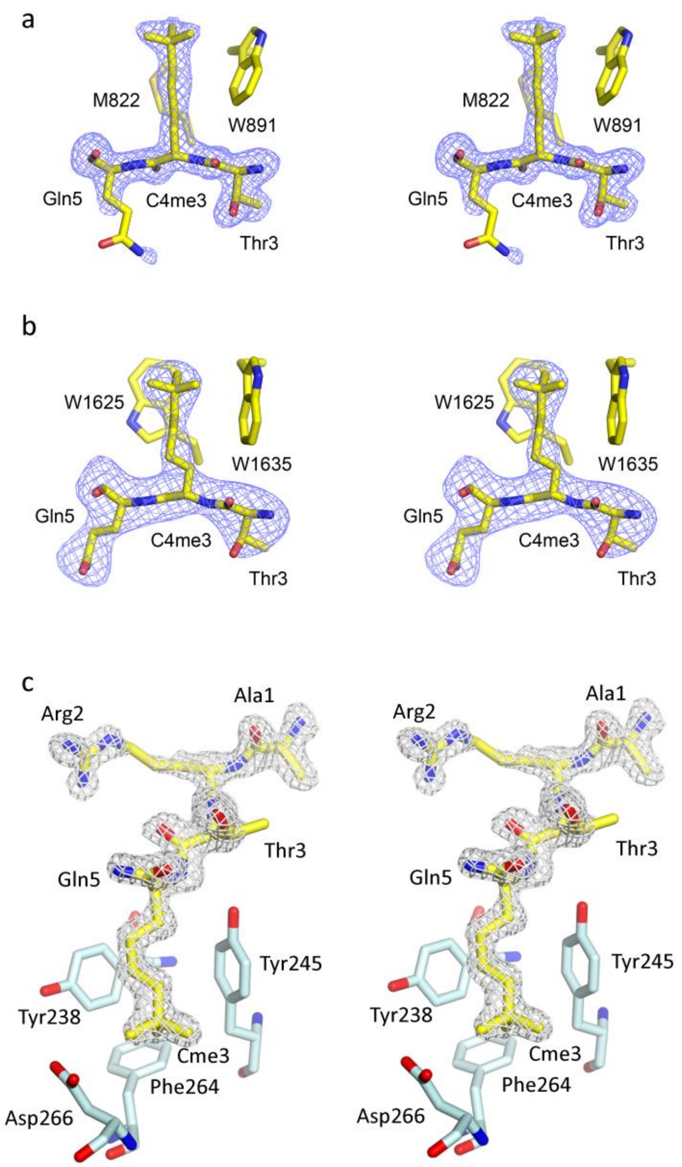

Supplementary Figure 2. Electron density map, displayed as blue or white mesh, for H3C4me3 in the reader–H3C4me3 complexes. (**a**) TAF3–H3C4me3 (Fo-Fc electron density contoured to 2.5σ, T3-C4-Q5 residues omitted for map production); (**b**) JARID1A–H3C4me3 (Fo-Fc electron density contoured to 1.5σ, residues T3-C4-Q5 omitted for map production, a sharpening B-factor of 60 is applied); (**c**) SGF29–H3C4me3 (Fo-Fc electron density contoured to 3σ).

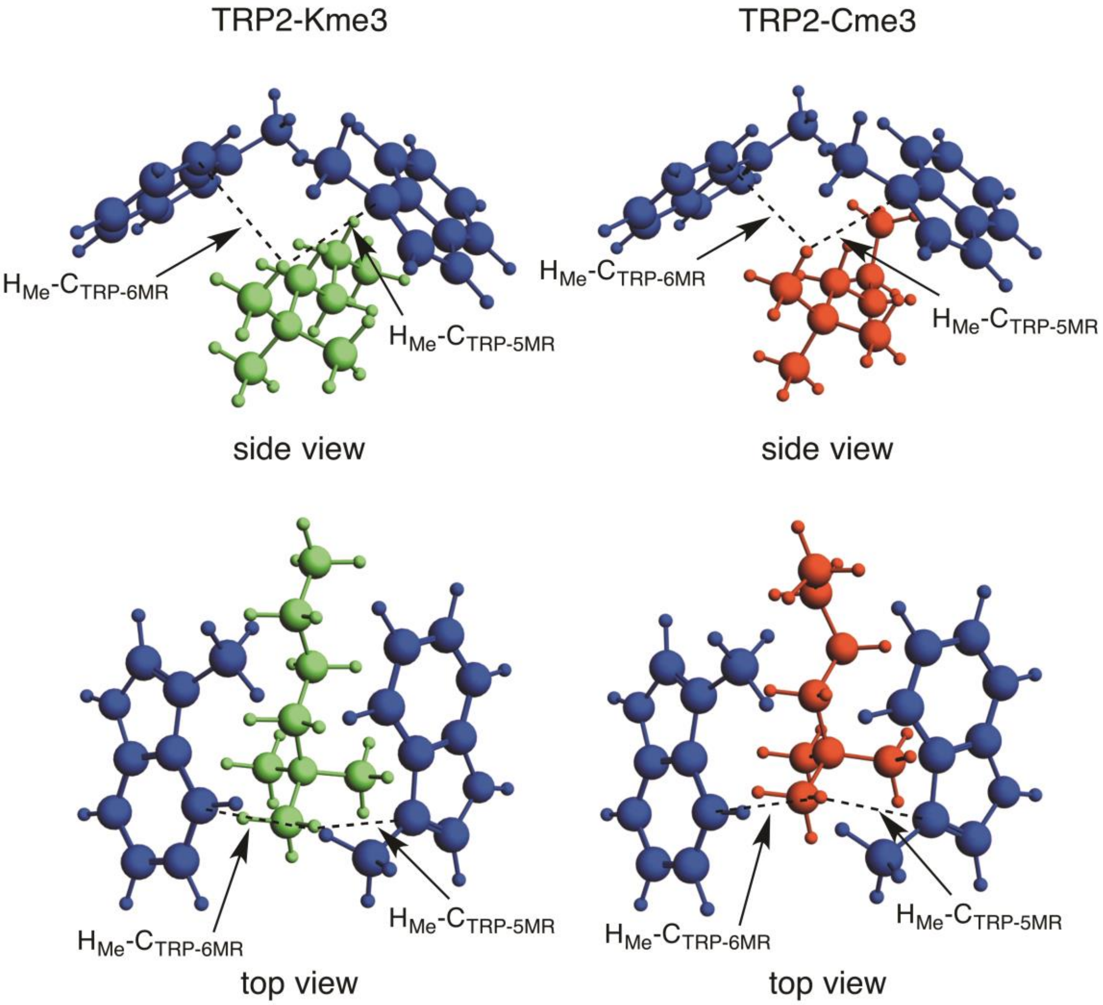

Supplementary Figure 3. Structures of JARID1A-Kme3 and JARID1A-Cme3 model complexes. TRP2 in blue, Kme3 in green and Cme3 in red.

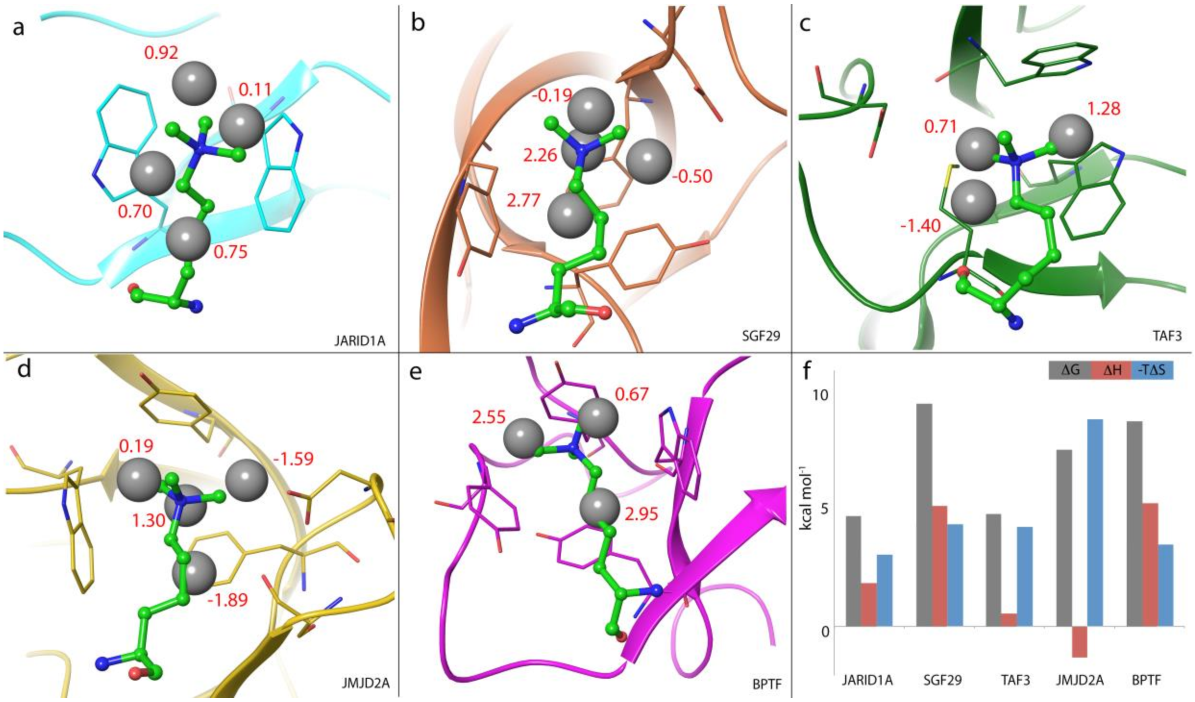

Supplementary Figure 4. WaterMap calculations for the solvation enthalpies of aromatic cages of reader proteins. (**a**) JARID1A; (**b**) SGF29; (**c**) TAF3; (**d**) JMJD2A; (**e**) BPTF. Superimposed Kme3 side chain and water molecules are presented as green stick and grey spheres. Numbers (red) adjacent to grey spheres represent the value of the enthalpy (Δ*H*) for individual water molecule; (**f**) Thermodynamic parameters for the solvation of the aromatic cages of five reader proteins.

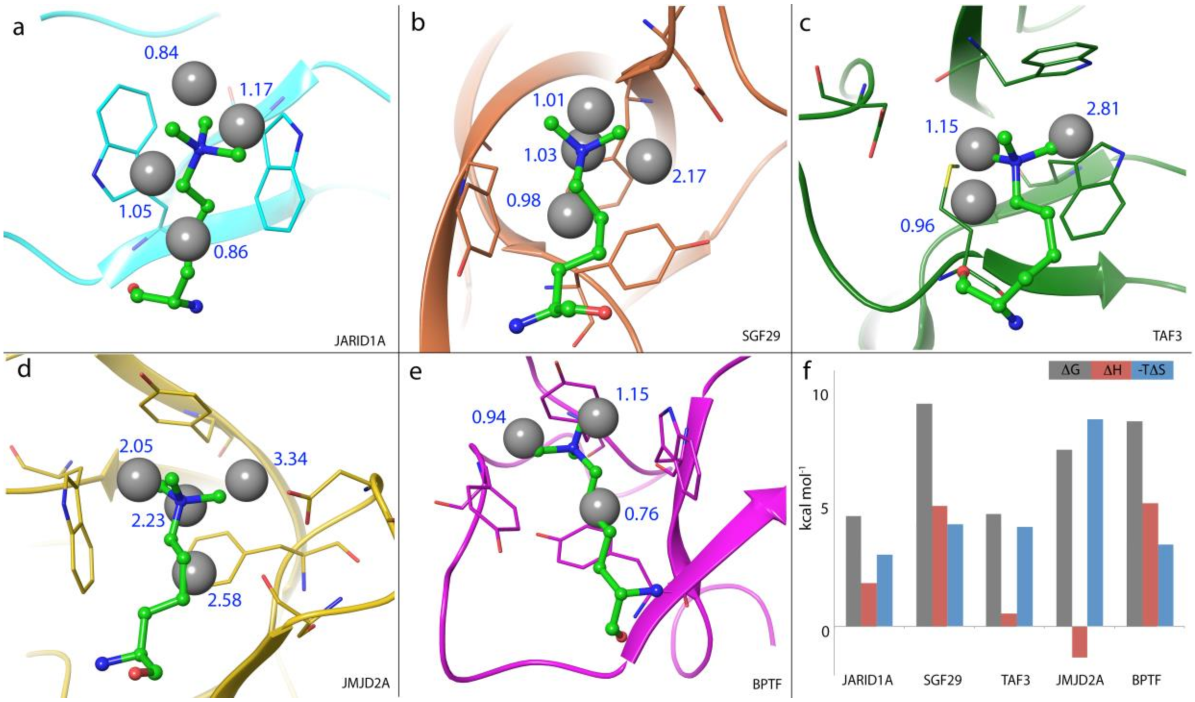

Supplementary Figure 5. WaterMap calculations for the solvation entropies of aromatic cages of reader proteins. (**a**) JARID1A; (**b**) SGF29; (**c**) TAF3; (**d**) JMJD2A; (**e**) BPTF. Superimposed Kme3 side chain and water molecules are presented as green stick and grey spheres. Numbers (blue) adjacent to grey spheres represent the value of the entropy (–TΔS) for individual water molecule; (**f**) Thermodynamic parameters for the solvation of the aromatic cages of five reader proteins.

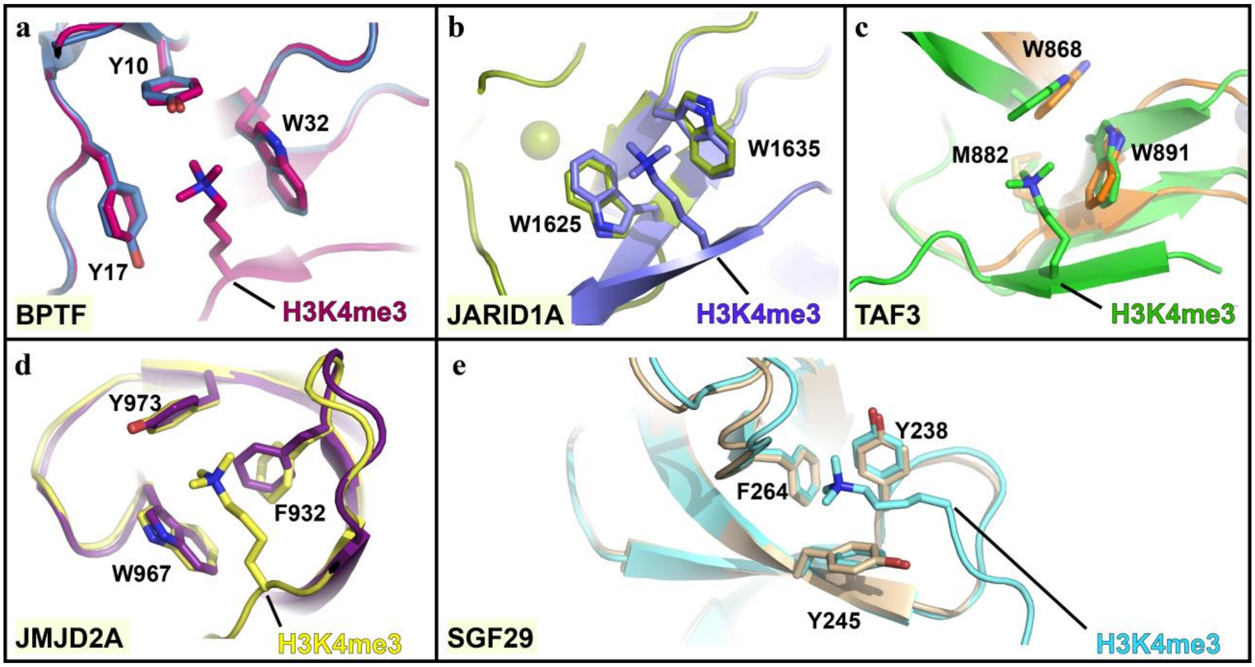

Supplementary Figure 6. Comparison of free (unbound, apo) aromatic cages and Kme3-bound (holo) aromatic cages illustrates that aromatic cages are predominantly preformed for binding of the Kme3 substrate. (**a**) BPTF PHD finger; (**b**) JARID1A PHD finger; (**c**) TAF3 PHD finger; (**d**) JMJD2A Tudor; (**e**) SGF29 Tudor. Coordinates are taken from Protein Data Bank with entry numbers 2F6N (free) and 2F6J (complex) for BPTF; 2KGG (free) and 3GL6 (complex) for JARID1A; 2K16 (free) and 2K17 (complex) for TAF3; 2GF7 (free) and 2GFA (complex) for JMJD2A; 3LX7 (free) and 3MEA (complex) for SGF29, respectively.

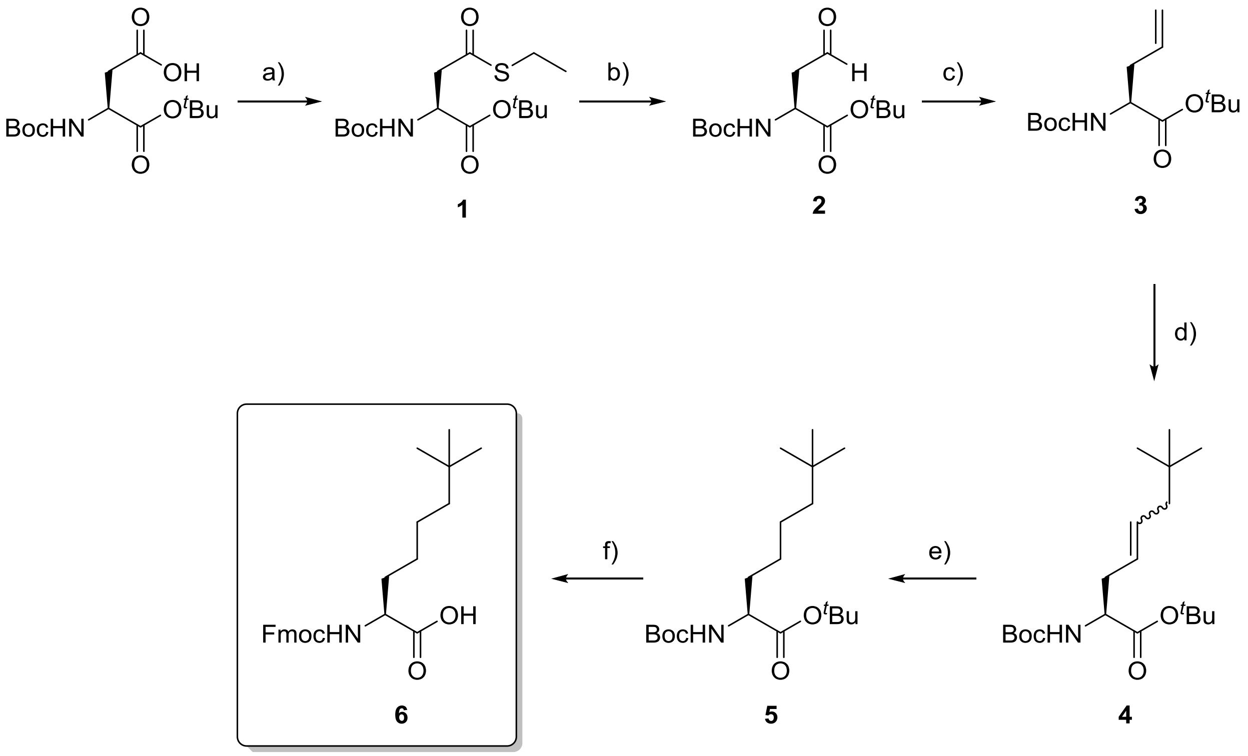

Supplementary Figure 7. Synthetic scheme for the preparation of Fmoc-L-Cme3 (6).

Conditions: a) DMAP, DCC, EtSH, CH_2Cl_2, 94%; b) 10% Pd/C, Et_3SiH, CH_2Cl_2, 95%; c) methyl triphenyl phosphonium bromide, NaHMDS, CH_2Cl_2, 60%; d) 4,4-dimethylpent-1-ene, 2nd generation Grubbs catalyst, CH_2Cl_2, 50 °C, 56%; e) 10% Pd/C, $H_{2\,(g)}$, CH_2Cl_2, 95%; f) 1) TFA, CH_2Cl_2, 2) Fmoc-OSu, $NaHCO_3$, H_2O : 1,4-dioxane (1 : 1), 84%

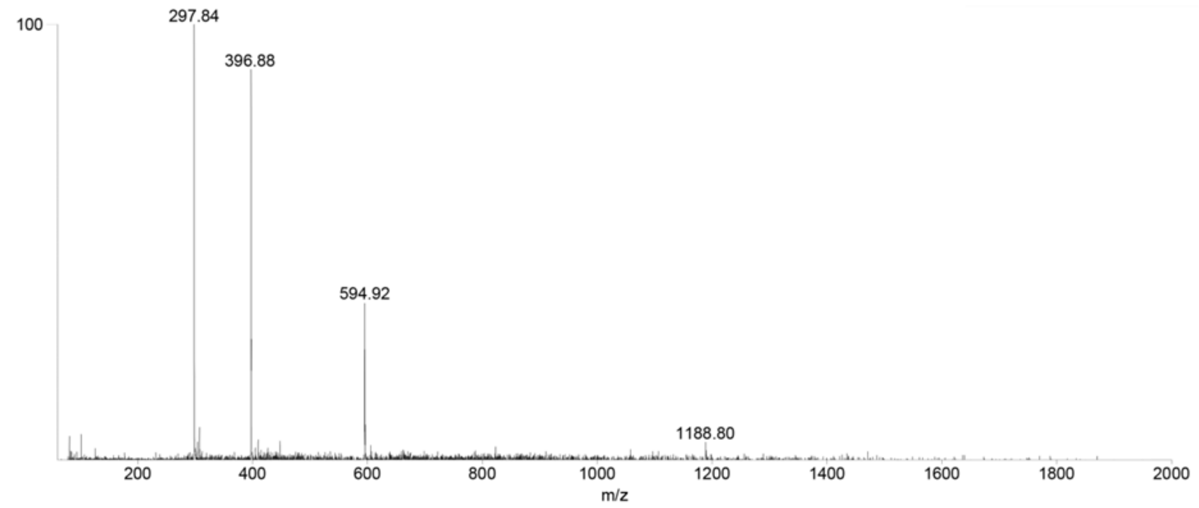

Supplementary Figure 8. ESI-MS data of the 10-mer H3K4me3 peptide. 1188.80 [M^{+}], 594.92 [M^{2+}], 396.88 [M^{3+}], 297.84 [M^{4+}].

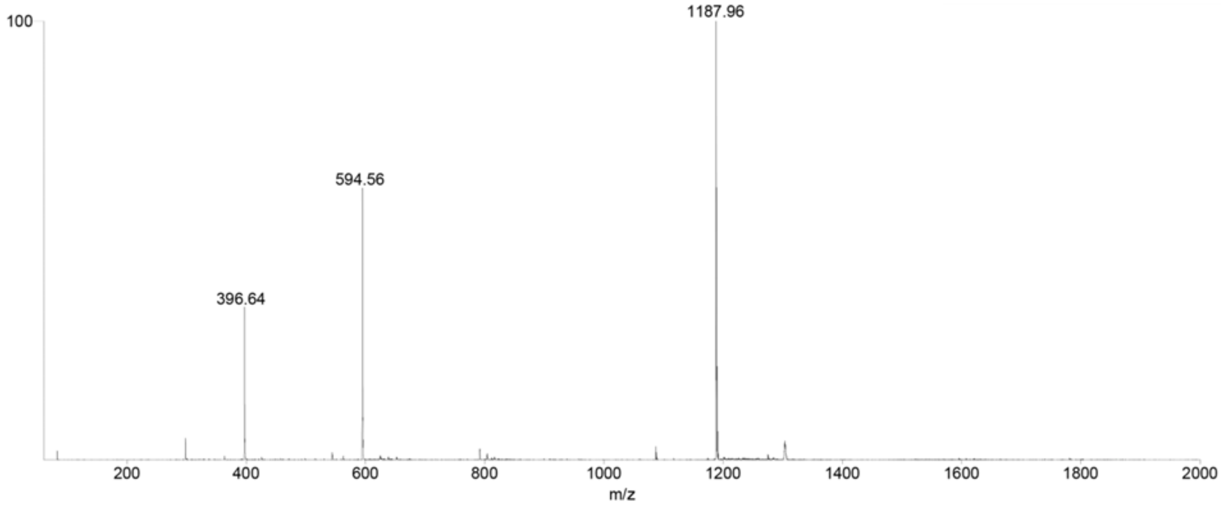

Supplementary Figure 9. ESI-MS data of the 10-mer H3C4me3 peptide. 1187.96 [M^{+}], 594.56 [M^{2+}], 396.64 [M^{3+}], 297.6 [M^{4+}].

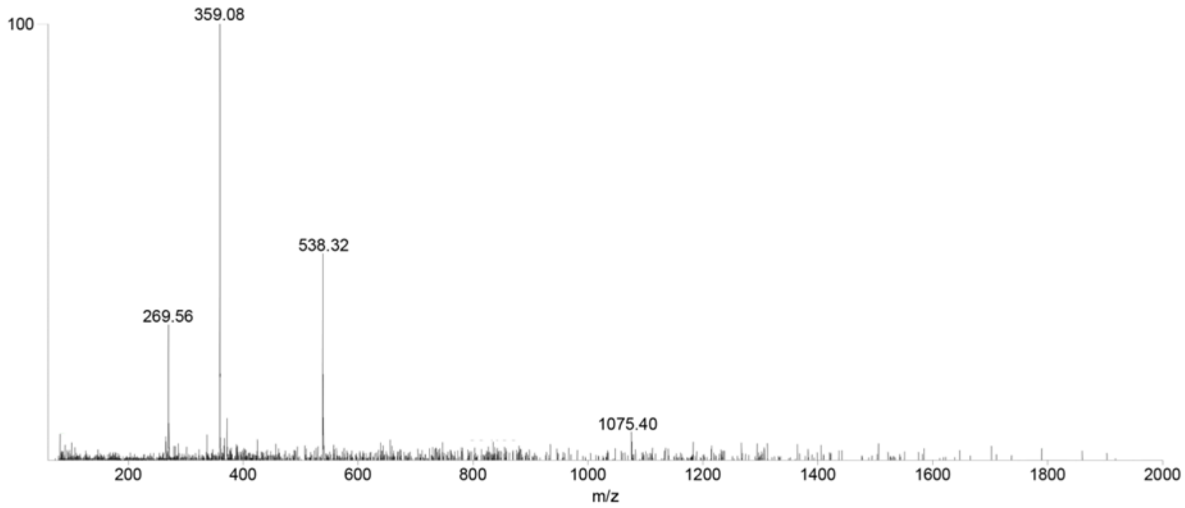

Supplementary Figure 10. ESI-MS data of the 10-mer H3G4 peptide. 1075.40 [M^+], 538.32 [M^{2+}], 359.08 [M^{3+}], 269.56 [M^{4+}].

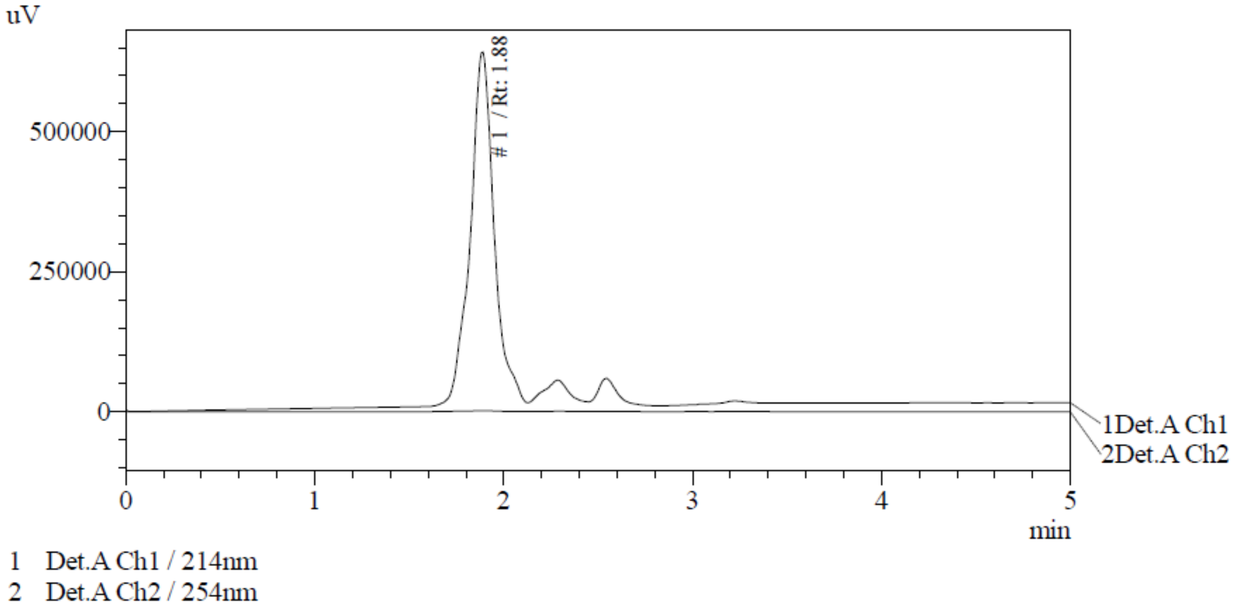

Supplementary Figure 11. HPLC trace data of the 10-mer H3K4me3 peptide. Peaks at 2.5 minutes derive from injection (blank).

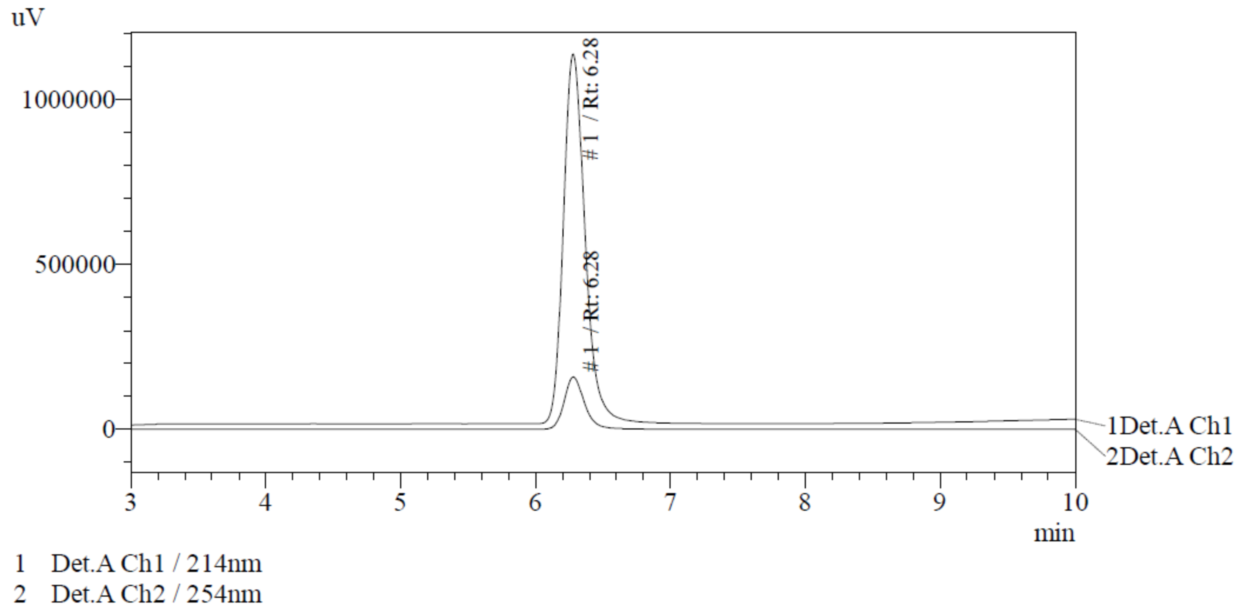

Supplementary Figure 12. HPLC trace analysis of 10-mer H3C4me3 peptide.

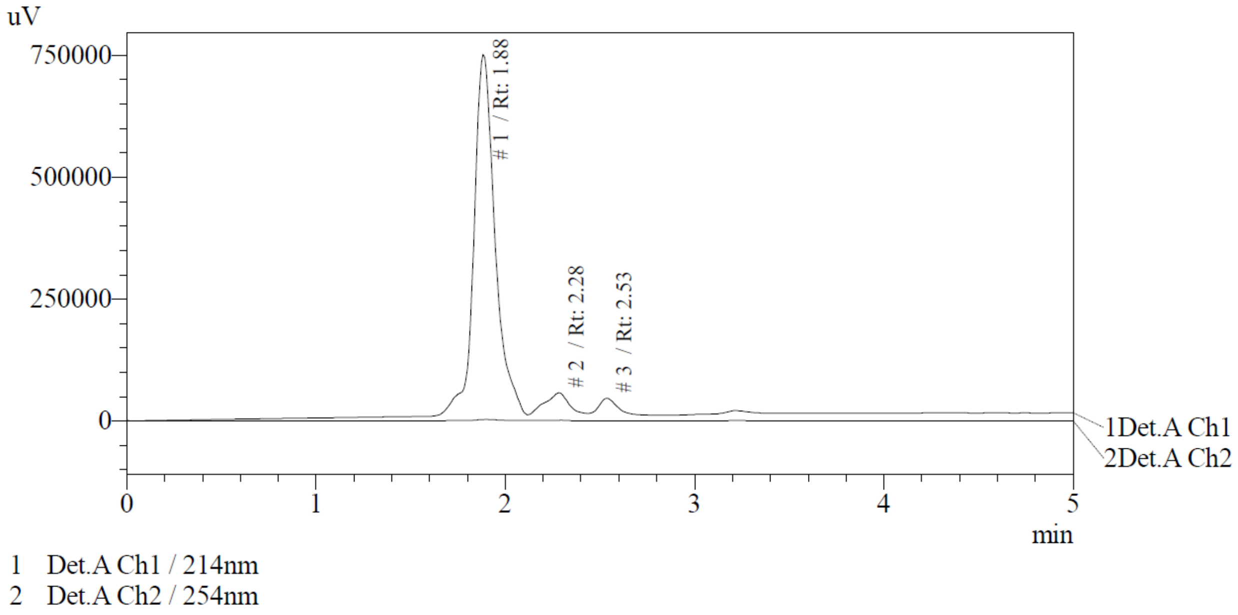

Supplementary Figure 13. HPLC trace analysis of 10-mer H3G4 peptide. Peaks at 2.5 minutes derive from injection (blank).

Supplementary Table 1. Cartesian coordinates of TRP2-Kme3 and TRP2-Cme3 systems, computed at BLYP-D3BJ/TZ2P using COSMO to simulate aqueous solvation and a constrained optimization to simulate the effect of the protein backbone.

TRP2-Kme3:

C	-14.114000000	-20.049000000	-0.875000000
C	-14.962000000	-19.738000000	0.323000000
C	-15.235000000	-20.561000000	1.377000000
C	-15.571000000	-18.476000000	0.628000000
C	-16.191000000	-18.610000000	1.893000000
C	-15.649000000	-17.250000000	-0.044000000
N	-15.971000000	-19.886000000	2.326000000
C	-16.882000000	-17.550000000	2.500000000
C	-16.335000000	-16.198000000	0.561000000
C	-16.943000000	-16.358000000	1.823000000
H	-17.473000000	-15.517000000	2.270000000
H	-14.000000000	-19.128000000	-1.447000000
H	-14.917000000	-21.601000000	1.456000000
H	-15.183000000	-17.121000000	-1.021000000
H	-16.295000000	-20.273000000	3.201000000
H	-17.354000000	-17.669000000	3.475000000
H	-16.402000000	-15.237000000	0.051000000
H	-13.186000000	-20.452000000	-0.470000000
C	-13.008000000	-14.944000000	-1.752000000
C	-11.604000000	-15.279000000	-1.421000000
C	-10.629000000	-14.423000000	-0.994000000
C	-10.999000000	-16.571000000	-1.507000000
C	-9.651000000	-16.428000000	-1.114000000
C	-11.469000000	-17.840000000	-1.880000000
N	-9.451000000	-15.109000000	-0.805000000
C	-8.764000000	-17.507000000	-1.084000000
C	-10.588000000	-18.912000000	-1.851000000
C	-9.247000000	-18.738000000	-1.453000000
H	-8.579000000	-19.599000000	-1.438000000
H	-13.651000000	-15.747000000	-1.391000000
H	-10.764000000	-13.354000000	-0.828000000
H	-12.506000000	-17.981000000	-2.186000000
H	-8.581000000	-14.705000000	-0.490000000
H	-7.726000000	-17.376000000	-0.779000000
H	-10.938000000	-19.903000000	-2.140000000
H	-13.236000000	-13.992000000	-1.272000000
H	-14.522374582	-20.815706247	-1.547885097
H	-13.158423449	-14.818069475	-2.834003080

C	-10.114752602	-21.305220763	1.892377384
C	-11.216790553	-20.285805453	1.565697139
C	-11.002507694	-18.940388922	2.287874794
C	-12.090102444	-17.946438531	1.883398917
N	-12.070579136	-16.609009105	2.645799587
C	-13.150209827	-15.721875154	2.061139245
C	-10.731973677	-15.916856909	2.492497579
C	-12.365551231	-16.823749860	4.115733524
H	-12.408951734	-15.846708144	4.598931529
H	-9.130774938	-20.927740553	1.584711029
H	-12.198761989	-20.691389652	1.844793551
H	-10.013332154	-18.548608026	2.021388165
H	-13.089556823	-18.360771942	2.048366284
H	-12.918787327	-15.550085307	1.009793743
H	-10.798479096	-14.941374934	2.976931329
H	-11.570405162	-17.423783848	4.555007649
H	-10.074709060	-21.512398227	2.969877086
H	-11.238229637	-20.105747697	0.484787617
H	-11.011890000	-19.111207919	3.371339633
H	-11.995495315	-17.683123359	0.825941434
H	-13.153012338	-14.778635478	2.609325685
H	-10.522561113	-15.800602201	1.428638506
H	-13.326128627	-17.334212341	4.203429545
H	-14.111323996	-16.226695834	2.162298263
H	-9.963428736	-16.520728269	2.972373589
H	-10.290718944	-22.253949886	1.371226478

TRP2-Cme3:

C	45.969000000	21.413000000	-4.047000000
C	47.156000000	21.171000000	-4.864000000
C	47.384000000	21.634000000	-6.121000000
C	48.352000000	20.472000000	-4.468000000
C	49.246000000	20.533000000	-5.547000000
C	48.745000000	19.803000000	-3.313000000
N	48.632000000	21.237000000	-6.550000000
C	50.512000000	19.945000000	-5.503000000
C	50.004000000	19.224000000	-3.277000000
C	50.867000000	19.300000000	-4.360000000
H	51.850000000	18.833000000	-4.293000000
H	46.179000000	21.047000000	-3.042000000
H	46.683000000	22.231000000	-6.704000000
H	48.077000000	19.736000000	-2.454000000
H	49.030000000	21.433000000	-7.457000000
H	51.191000000	20.001000000	-6.354000000
H	50.323000000	18.697000000	-2.378000000

H	45.791000000	22.486000000	-4.112000000
C	48.458000000	21.019000000	0.442000000
C	48.203000000	22.440000000	0.724000000
C	48.923000000	23.255000000	1.539000000
C	47.166000000	23.243000000	0.161000000
C	47.321000000	24.540000000	0.673000000
C	46.133000000	22.992000000	-0.739000000
N	48.402000000	24.523000000	1.513000000
C	46.469000000	25.580000000	0.327000000
C	45.294000000	24.026000000	-1.087000000
C	45.465000000	25.305000000	-0.550000000
H	44.779000000	26.100000000	-0.840000000
H	48.122000000	20.804000000	-0.572000000
H	49.787000000	22.946000000	2.127000000
H	45.992000000	21.996000000	-1.159000000
H	48.758000000	25.315000000	2.029000000
H	46.599000000	26.579000000	0.742000000
H	44.483000000	23.845000000	-1.793000000
H	49.529000000	20.858000000	0.570000000
H	47.917587245	20.339731840	1.117950276
H	45.074268746	20.888635016	-4.411285300
C	45.938673402	25.400169396	-4.801600761
C	47.219291385	26.197067425	-5.110059086
C	48.318339411	26.067679959	-4.033166971
C	48.803467145	24.620565562	-3.798529201
C	50.163863734	24.473911953	-3.054136190
C	50.393189646	22.976715818	-2.753234187
C	50.146689730	25.252287263	-1.718848139
C	51.324179650	24.987056753	-3.940305728
H	51.220417800	26.055260778	-4.170687694
H	46.142673849	24.324644598	-4.789001017
H	46.964295557	27.260421539	-5.229267853
H	47.938259503	26.479210225	-3.086664777
H	48.890275509	24.102887082	-4.767449173
H	50.390606339	22.383603674	-3.675538007
H	50.051173620	26.333514434	-1.882223403
H	51.360888027	24.436224541	-4.890836872
H	45.534381872	25.668036767	-3.816507433
H	47.626397082	25.864759449	-6.077601665
H	49.164222311	26.702316747	-4.332341408
H	48.040950132	24.073164023	-3.227425802
H	49.603713613	22.584404511	-2.101863226
H	51.078688756	25.075638238	-1.163210023
H	52.288303084	24.848278156	-3.431025882
H	51.357977767	22.826588619	-2.247751921
H	49.309463792	24.924546312	-1.092088497

H	45.162492112	25.585522346	-5.555364852

Supplementary Table 2. Overlaps between the MOs of TRP and either Kme3 or Cme3.[a]

**TRP MOs**	**Kme3/Cme3 MOs**	**JARID1A-Kme3**	**JARID1A-Cme3**
HOMO	LUMO	0.012	0.002
HOMO	LUMO+1	0.006	0.009
HOMO-1	LUMO	0.028	0.015
HOMO-1	LUMO+1	0.006	0.000

[a] Computed at BLYP-D3BJ/TZ2P.

Supplementary Table 3. WaterMap calculations of thermodynamic parameters (in kcal mol^{-1}) for the solvation of aromatic cages of five reader proteins. Calculations are based on the crystal structures of the apo form of reader proteins or holo form of reader proteins. Structures of reader proteins are obtained from PDB (see PDB IDs for apo and holo forms).

	Apo	$-T\Delta S$	ΔH	ΔG	Holo	Kme3	$-T\Delta S$	ΔH	ΔG
JARID1A	2KGG	1.4	0.8	2.2		2KGI	2.8	1.7	4.3
SGF29	3MEW	2.9	6.8	9.7		3ME9	4.0	4.7	8.7
TAF3	2K16	1.6	2.5	4.1		2K17	3.9	0.5	4.4
JMJD2A	2GF7	6.0	-0.8	5.2		2GFA	8.1	-1.2	6.9
BPTF	2F6N	3.5	3.6	7.1		2F6J	3.2	4.8	8
